# Cerenkov luminescence imaging and flexible autoradiography for specimen margin assessment during breast-conserving cancer surgery

**DOI:** 10.1093/radadv/umae015

**Published:** 2024-05-24

**Authors:** Aaditya Sinha, Zhane Peterson, Belul Shifa, Hannah Jeffery, Patriek Jurrius, Sarah Allen, Eugene Lee, Mohammed Azmat, Rachel Barrass, Damion Bailey, Jessica Johnson, Kathryn Adamson, Vasileios Karydakis, Elina Shaari, Mangesh Thorat, Hisham Hamed, Georgina Bitsakou, Sarah Pinder, Padma Menon, Wen Ng, Gary Cook, John Joemon, Armidita Jacob, Sofia Pereira, Jocelyn Thomas, Ruheana Begum, Karim El-Boghdadly, Mieke Van Hemelrijck, Ashutosh Kothari, Arnie Purushotham

**Affiliations:** King’s College London, London SE1 9RT, United Kingdom; Guy's and St Thomas’ NHS Foundation Trust, London SE1 9RT, United Kingdom; King’s College London, London SE1 9RT, United Kingdom; Guy's and St Thomas’ NHS Foundation Trust, London SE1 9RT, United Kingdom; King’s College London, London SE1 9RT, United Kingdom; Guy's and St Thomas’ NHS Foundation Trust, London SE1 9RT, United Kingdom; King’s College London, London SE1 9RT, United Kingdom; Guy's and St Thomas’ NHS Foundation Trust, London SE1 9RT, United Kingdom; King’s College London, London SE1 9RT, United Kingdom; Guy's and St Thomas’ NHS Foundation Trust, London SE1 9RT, United Kingdom; King’s College London, London SE1 9RT, United Kingdom; Guy's and St Thomas’ NHS Foundation Trust, London SE1 9RT, United Kingdom; Guy's and St Thomas’ NHS Foundation Trust, London SE1 9RT, United Kingdom; Guy's and St Thomas’ NHS Foundation Trust, London SE1 9RT, United Kingdom; Guy's and St Thomas’ NHS Foundation Trust, London SE1 9RT, United Kingdom; Guy's and St Thomas’ NHS Foundation Trust, London SE1 9RT, United Kingdom; Guy's and St Thomas’ NHS Foundation Trust, London SE1 9RT, United Kingdom; Guy's and St Thomas’ NHS Foundation Trust, London SE1 9RT, United Kingdom; Guy's and St Thomas’ NHS Foundation Trust, London SE1 9RT, United Kingdom; Guy's and St Thomas’ NHS Foundation Trust, London SE1 9RT, United Kingdom; Guy's and St Thomas’ NHS Foundation Trust, London SE1 9RT, United Kingdom; Guy's and St Thomas’ NHS Foundation Trust, London SE1 9RT, United Kingdom; Guy's and St Thomas’ NHS Foundation Trust, London SE1 9RT, United Kingdom; King’s College London, London SE1 9RT, United Kingdom; Guy's and St Thomas’ NHS Foundation Trust, London SE1 9RT, United Kingdom; Guy's and St Thomas’ NHS Foundation Trust, London SE1 9RT, United Kingdom; Guy's and St Thomas’ NHS Foundation Trust, London SE1 9RT, United Kingdom; King’s College London, London SE1 9RT, United Kingdom; Guy's and St Thomas’ NHS Foundation Trust, London SE1 9RT, United Kingdom; Guy's and St Thomas’ NHS Foundation Trust, London SE1 9RT, United Kingdom; Guy's and St Thomas’ NHS Foundation Trust, London SE1 9RT, United Kingdom; Guy's and St Thomas’ NHS Foundation Trust, London SE1 9RT, United Kingdom; Guy's and St Thomas’ NHS Foundation Trust, London SE1 9RT, United Kingdom; Guy's and St Thomas’ NHS Foundation Trust, London SE1 9RT, United Kingdom; King’s College London, London SE1 9RT, United Kingdom; Guy's and St Thomas’ NHS Foundation Trust, London SE1 9RT, United Kingdom; King’s College London, London SE1 9RT, United Kingdom; Guy's and St Thomas’ NHS Foundation Trust, London SE1 9RT, United Kingdom; King’s College London, London SE1 9RT, United Kingdom; Guy's and St Thomas’ NHS Foundation Trust, London SE1 9RT, United Kingdom; King’s College London, London SE1 9RT, United Kingdom; Guy's and St Thomas’ NHS Foundation Trust, London SE1 9RT, United Kingdom

**Keywords:** breast cancer, breast-conserving surgery, intra-operative novel imaging, Cerenkov luminescence, flexible autoradiography

## Abstract

**Background:**

Among women with breast cancer who undergo breast-conserving surgery (BCS), 20% to 25% require further surgery because of close or involved margins. Improved techniques are needed to assess resection margins.

**Purpose:**

The study aims were to assess the feasibility of the combined techniques of Cerenkov luminescence imaging–flexible autoradiography (CLI-FAR) to assess excision specimen margins in women undergoing BCS and to determine the diagnostic performance of intraoperative CLI-FAR imaging with postoperative histopathology as the reference standard.

**Materials and Methods:**

Women undergoing BCS were recruited prospectively at a single center over 13 months. Patients were injected with 250 MBq ± 10 MBq of 18F-fluorodeoxyglucose, 145 minutes before surgery; the excised specimens were imaged intraoperatively. The surgically excised tumor was initially imaged using conventional x-ray, and margins suspected to be involved by tumor were then imaged using CLI-FAR. CLI-FAR imaging was performed using the LightPath system (Lightpoint), an in vitro diagnostic device designed to identify and locate positron-emitting radionuclides. Any suspicious margin underwent an immediate reexcision in the form of cavity shavings. Sensitivity, specificity, and positive and negative predictive values while considering histopathological assessment as the golden standard were used to assess the performance of CLI-FAR.

**Results:**

In all, 54 specimens were imaged in 52 patients, with a total of 104 margins reviewed using CLI-FAR. The results showed a specificity of 97.8% (89/91; 95% confidence interval [CI], 95.0-100.6), sensitivity of 76.9% (10/13; 95% CI, 68.3-85.0), positive predictive value of 83.3% (10/12; 95% CI, 76.2-90.5), and negative predictive value of 96.7% (89/92; 95% CI, 93.3-100.2). In all, 8 patients had 10 positive margins on CLI-FAR imaging and were treated accordingly. CLI-FAR imaging reduced the reexcision rate by 69% (17.3/25).

**Conclusion:**

CLI-FAR imaging is a promising technique for intraoperative margin assessment in women undergoing BCS for invasive breast cancer.

**Summary**

Cerenkov luminescence imaging–flexible autoradiography is a novel technique that shows promise for reducing the reexcision rate by assessing intraoperative margins during breast-conserving surgery for invasive breast cancer.

**Key Results**

- Intraoperative Cerenkov luminescence imaging–flexible autoradiography (CLI-FAR) can decrease the reexcision rate in breast-conserving surgery for invasive cancer by up to 69% (17.3/25).
- Intraoperative margin assessment using CLI-FAR during breast-conserving surgery for invasive cancer showed a specificity of 97.8% (89/91) and sensitivity of 76.9% (10/13).
- Mean delay between surgical excision and CLI-FAR images was 6 minutes, indicating CLI-FAR is feasible for use in hospitals without disrupting standard practice or causing significant delays in the operating room.

## Introduction

Women diagnosed with breast cancer undergo breast-conserving surgery (BCS) or mastectomy (± reconstruction) for the primary tumor. Alternatively, patients receive neoadjuvant chemotherapy (NACT), followed by BCS.^1^ Approximately 70% of women undergo BCS. However, around 20% to 25% of patients who undergo BCS require further excision because positive margins.^2–5^ In the United Kingdom, institutions follow the Association of Breast Surgeon Guidelines,^6^ with a positive margin indicated if the tumor is found in the inked edge of the specimen, within 1 mm for invasive or ductal carcinoma in situ (DCIS) associated with invasive disease, or within 2 mm for pure DCIS, whereas other institutions call a positive margin as tumor on ink. Despite variations worldwide in the definition of positive margin,^5^^,^^7^^,^^8^ overall, positive margins increase the risk of local recurrence with potential increased risk of distant recurrence and death.^7–9^ As such, reexcision is recommended.

Additional surgery may result in poorer cosmesis and increased psychological morbidity and costs for the patient and health care system.^5^^,^^10^ Techniques identified for intraoperative margin assessment are shown in Table S1. However, these are not used widely. Because of their low sensitivity, specificity, and high cost,^2^^,^^11^ only intraoperative radiography in BCS has become the standard of care internationally.^12–14^ Therefore, innovative techniques are required.

Cerenkov luminescence imaging–flexible autoradiography (CLI-FAR) is a novel dual-modality imaging method for detecting cancer cell radioactivity using optical and molecular imaging. For molecular imaging, 18F-fluorodeoxyglucose (^18^F-FDG) is used.^15^ These modalities have been individually investigated in BCS margin assessment.^16^^,^^17^ CLI detects Cerenkov luminescence directly. This luminescence is generated as a faint blue light when a charged particle, such as a positron, moves through a medium at a speed greater than light.^15^ The imaging modality uses real-time imaging, which includes advantages from optical white light and positron emission tomography imaging. FAR indirectly detects scintillations caused by charged particles like positrons exciting a thin scintillating film. The advantage of using a scintillator is that it ensures that only charged particles can produce scintillations, eliminating any diathermy artifact in FAR.

The primary aims of this study were to assess the feasibility of CLI-FAR to assess excision margins in women undergoing BCS and to determine the diagnostic performance of intraoperative CLI-FAR imaging with postoperative histopathology as the reference standard. The secondary objectives were to compare the margin status of specimens obtained during BCS using CLI-FAR and routine specimen x-rays, reoperation rates, and assess additional surgical time.

## Material and methods

### Clinical trial setup

A single‐arm interventional first-in-human study was designed to evaluate the diagnostic accuracy of CLI-FAR in conjunction with ^18^F-FDG to assess tumor margins in BCS. The study was approved by a UK independent ethics committee (REC15/LO/0029), the Administration of Radioactive Substances Advisory Committee, and the Health Research Authority (IRAS314460) (ClinicalTrials.gov identifier: NCT05496101). All the documents submitted to these regulatory bodies detailed the intraoperative intervention in patients with a positive margin on CLI-FAR, and approvals were granted based on this information.

### Recruitment

Patients were identified during the Breast Multi-Disciplinary Meeting. Following written, informed consent, patients were recruited between November 2022 and December 2023 (Figures S1 and S2). The study checklist (Figure S3) was completed before the patient proceeded to the nuclear medicine department on the day of surgery (Figure S4).

Patients older than age 18 years with a diagnosis of invasive breast cancer undergoing BCS were included. Patients who have had surgery or radiotherapy to the ipsilateral breast in the past 12 months, known hypersensitivity to ^18^F-FDG, or who were pregnant or lactating were excluded.

### In vitro studies to minimize diathermy artifact

Diathermy artifact in CLI has been categorized as chemiluminescence^18^; this is a phenomenon of heat energy. Preclinical in vitro studies were conducted to distinguish between chemiluminescence and radioactivity to minimize the false-positive rate of signals obtained. That the brightness of chemiluminescence reduced with lower diathermy energy (W, watts) levels and time after exposure (Figure S5) was noted. Therefore, we chose to use a maximum diathermy level of 20 W.

### Radio-tracer administration

Patients were injected intravenously with 250 MBq ± 10% of ^18^F-FDG approximately 145 minutes before the expected time of imaging the excised specimen. This dose was based on the previous study by Grootendorst et al.^16^ Patients undergoing axillary lymph node dissection only received the ^18^F-FDG injection intravenously, whereas patients undergoing a sentinel lymph node biopsy received intravenous ^18^F-FDG, as part of the study and received up to 40 MBq of ^99m^Tc-albumin-nanocolloid (^99m^Tc) injected intradermally at the periareolar region on the ipsilateral breast, as the standard of care.

### Radiation safety

Previous studies with ^18^F-FDG in similar settings, with patients undergoing BCS and sentinel lymph node biopsy, have shown minimal exposure to radioactivity.^16^^,^^17^ Surgeons received a mean dose of 34 and 61.8 mSv, whereas anesthetists received a mean dose of 11 and 26.4 mSv over the 2 previous studies.^16^^,^^17^ UK legislation regarding the use of ionizing radiation was fully complied with.^19–21^ Staff members were provided personal body radiation dosimeters (MYDOSE mini, ALOKA, Mure, Mitaka-shi, Tokyo, Japan), and surgeons and anesthetists were provided with thermos-stimulated luminescent rings (Saturn TLD Rings, Landauer, Illinois, USA) to ensure radiation doses were monitored. All cases had dosimeters (41/44A, series 300 mini-monitor; Thermoscientific) measuring the activity of the room, equipment, staff, and waste. No additional measures were required for the ^18^F-FDG as its half-life (110 minutes) is shorter than of ^99m^Tc (362 minutes).^22^

### Surgery

After anesthetic induction, ^99m^Tc activity in the axilla was assessed using a gamma probe with a collimator to detect the sentinel node. In conditions with a weak signal of ^99m^Tc or a generalized high activity level in the axilla, patients were injected with a periareolar subcutaneous injection of Patent Blue V (Guerbert, France). Five surgical consultants were involved in the study, with each performing 150 breast operations on average per annum. The breast surgery was performed first before any axillary procedure. Surgeons used a scalpel or diathermy (BOWA ARC 303, BOWA Medical, UK) at a reduced energy level during excision. The excised specimen was orientated for histopathology as follows: 1 suture and clip for anterior, 2 sutures and clips for superior, and 3 sutures and clips in the direction of the nipple.

The excised specimen was initially imaged using the 3-dimensionalx-ray imaging system (Kubtec Mozart System, KUB Technologies, Stratford, CT, USA). If a margin was suspected to be close to the edge of the excised specimen clinically or on intraoperative x-ray, it was imaged using CLI-FAR. If no margins were deemed to be close/involved, the surgeon chose 2 margins that appeared to be the closest and assessed them.

On reviewing the images produced by CLI-FAR, the surgeon made a clinical decision whether to intervene surgically by immediately taking further tissue from the residual cavity (cavity shavings). If a specimen was too large for the scintillator, only a CLI image was taken and analyzed using 1 method. The surgeon’s interpretation was documented and compared to the final histopathology.

### Specimen analysis

**Figure 1.**
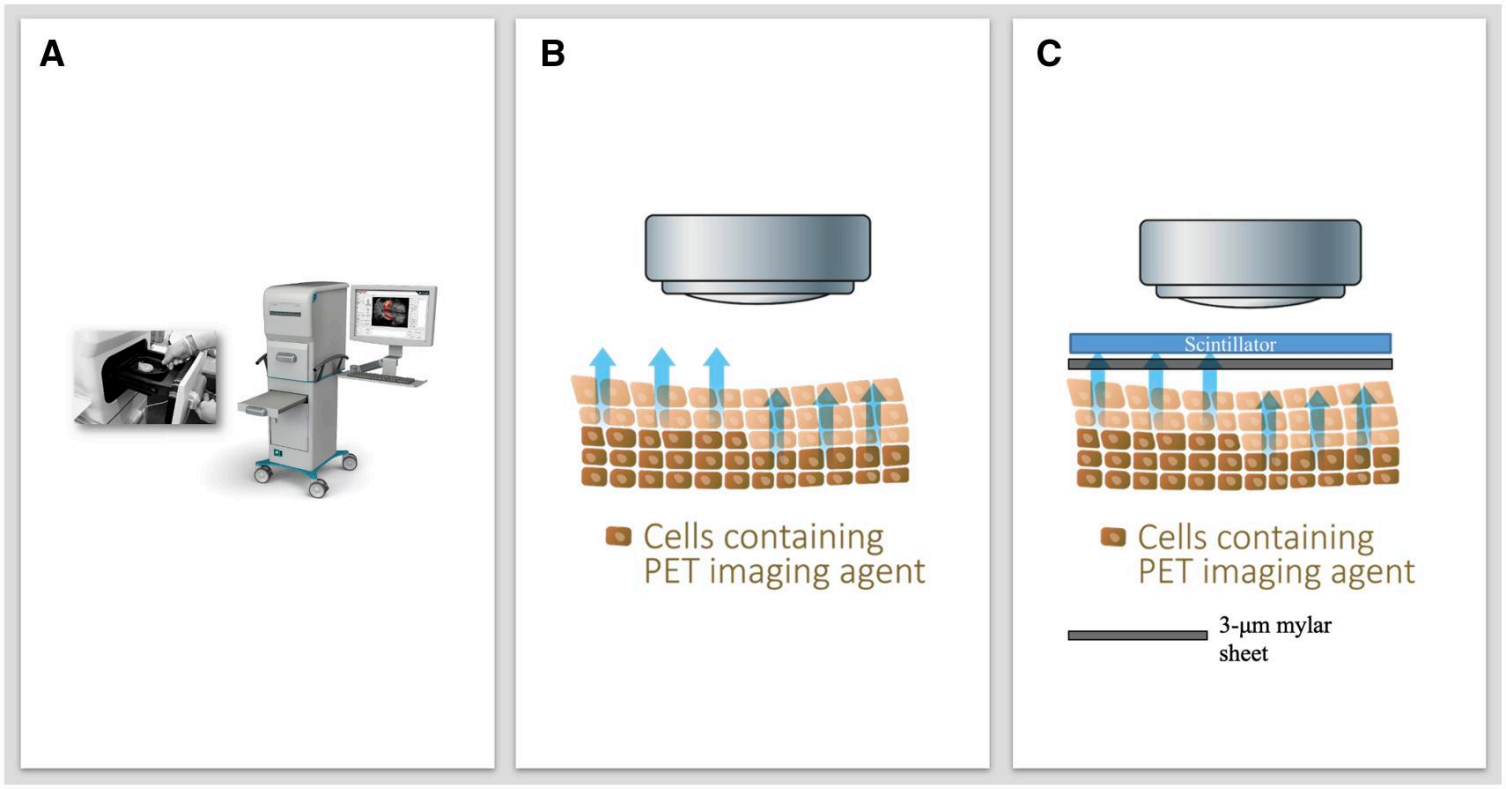
Imaging technology. (A) The LightPath System (Lightpoint Medical), (B) Cerenkov luminescence imaging (CLI), (C) flexible autoradiography (FAR) imaging: 3-μm Mylar sheet placed between scintillator and specimen. The specimen releasing beta particles is detected using an ultrasensitive camera for each imaging modality. Copyright granted by Lightpoint Medical for image in Figure 1A.

Both imaging techniques were obtained using the LightPath system (Lightpoint Medical Ltd, UK), an in vitro diagnostic device that detects the location and distribution of positron-emitting radionuclides within excised surgical specimens (Figure 1). The system is a bespoke device with an ultrasensitive camera that detects emitted activity between 550 and 850 nm. The Lightpath imaging system is not currently available commercially.

When examining tissue samples after surgery, CLI is a technique that uses noninvasive imaging to view tissues marked with a radiotracer (Figure 1). The LightPath system’s ultrasensitive camera detects the emitted light and creates an image of the tissue. Each margin must be separately imaged because the camera can only capture 1 2-dimentional image at a time.

When performing FAR, a 12-μm-thin scintillator was wrapped on the Wide Local Excision (WLE) specimen following Breast-Conserving Surgery (BCS), and a 3-μm Mylar sheet was placed between the specimen and scintillator to prevent contaminating the scintillator (Figure 1).^18^ To detect activity within a specific wavelength range of 550 nm ± 10%, a band path filter is used as the scintillator film produces scintillations in a limited wavelength range. The scintillator is white and completely opaque to chemiluminescence signatures.

It would take 30 to 60 seconds between images to wrap the scintillator and/or orientate the specimen. Both images had acquisition times of 300 seconds each and 8 × 8 pixel binning (total pixel resolution, 938 μm).

### Histopathology

Three histopathologists were blinded to the results of the CLI-FAR and x-ray imaging. All excised tissue specimens were examined for the presence of invasive or in situ disease, its size, and distance from all 6 margins, also reviewing the tumor type, grade, receptor status, presence or absence of vascular invasion, lymph node status, any additional molecular characteristics as requested by the multidisciplinary team to aid patient management, and the residual cancer burden if the patient had undergone NACT.

### Statistics

The sample size for this study was defined based on the assumption that this is a feasibility study. The primary endpoint of the study was to report the sensitivity and specificity of CLI and FAR LightPath imaging for tumor detection compared to positive tumor detection using standard-of-care histopathology methodology (positive margin of a WLE sample). Using an estimate of the incidence of positive excision margins on histopathology as 20%, with 95% confidence interval (alpha = 0.05 2-sided) and with 10% precision, a sample size of 54 patients or tumor specimens was estimated to provide sufficient power to detect sensitivity of 95% and specificity of 90%.^23^

Patient demographics and tumor characteristics as well as radiotracer administration and timing were reported with descriptive statistics. To assess the performance of CLI-FAR, we calculated sensitivity, specificity, and positive and negative predictive values while considering histopathological assessment as the golden standard. All statistical analyses were conducted in Stata (version 18.0, StataCorp LLC, College Station, TX).

The per-protocol population is defined as all patients who completed BCS and study procedures per protocol description. The primary endpoint was analyzed based on per protocol population.

## Results

**Figure 2.**
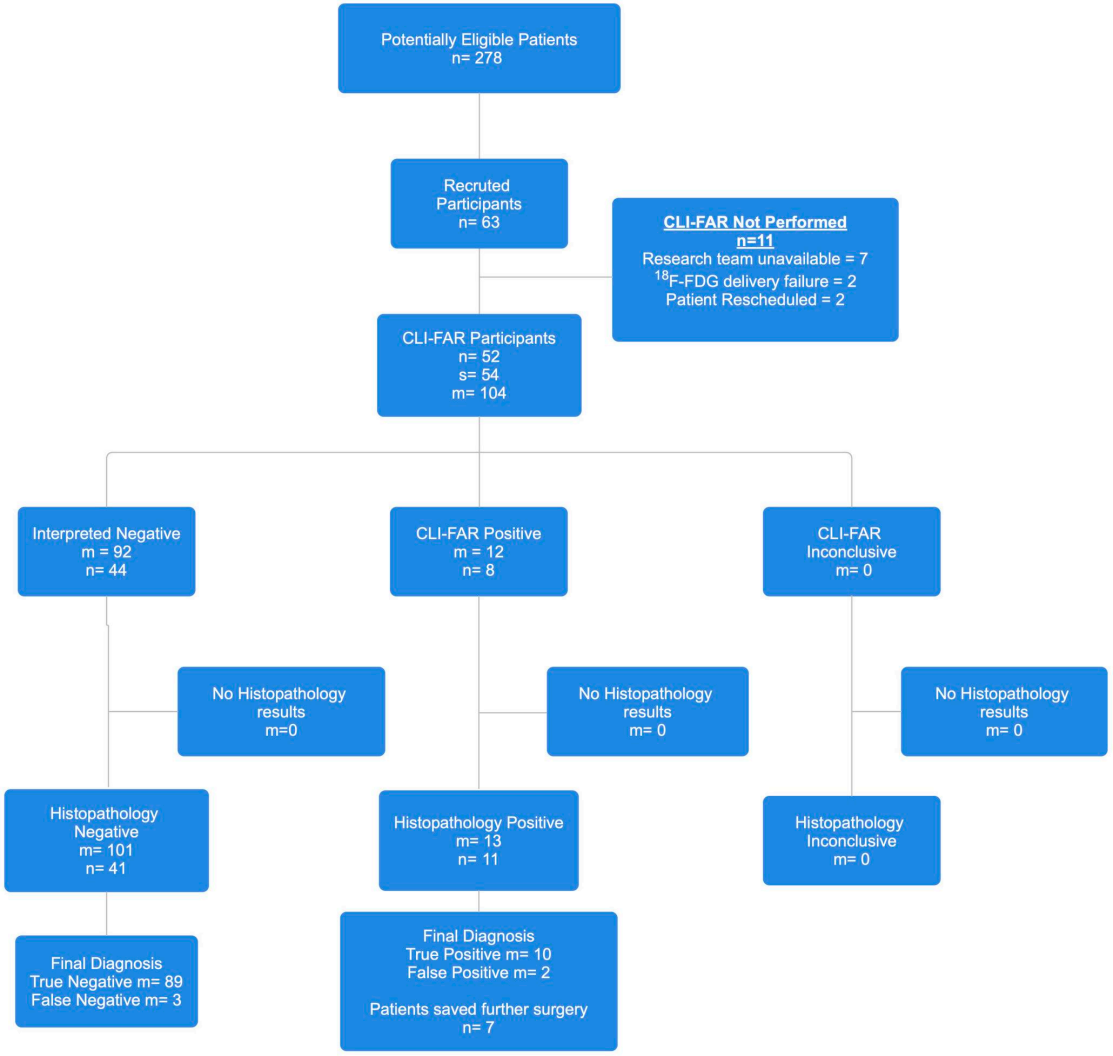
Patient and specimen flow. Abbreviations: M = number of margins; n = number of patients; s = number of samples.

Overall, 54 specimens were imaged in 52 patients with a total of 104 margins were reviewed using CLI-FAR (Figure 2). No adverse events were reported.

### Patient demographics and tumor characteristics

Demographic data for the patient and tumor characteristics are shown in Table 1 and Table S2. Of the 52 patients recruited with 54 tumors, 20 (38.5%) underwent NACT, with 11 (20.4%) tumors achieving a complete radiological response on magnetic resonance imaging before surgical resection. The remaining 43 tumors ranged in size (measured on preoperative magnetic resonance imaging, ultrasound, or mammogram) from 4 mm to 56 mm. The mean tumor size was 20.6 mm, excluding those with complete radiological response.

**Table 1.** Subject demographics and tumor features.

Patient demographics (n = 52)	
**Age (y)**	
20–44	9 (17.3%)
45–54	18 (34.6%)
55–64	14 (26.9%)
65+	11 (21.2%)
Mean (SD)	55.0 (12.6)
Range	22–84
**Treatment (n = 52)**	
Primary surgery	32 (61.5%)
Postneoadjuvant chemotherapy	20 (38.5%)
**Residual tumor burden (n = 20)**	
0	15 (75.0%)
2	3 (15.0%)
Not assessed/not reported	2 (10.0%)
**Tumor features (n = 54)**	
**Tumor size (mm)**	
0 (rCR)	11 (20.4%)
0–20	27 (50.0%)
21–50	15 (27.8%)
51+	1 (1.8%)
Mean (SD)	16.4 (13.3)
Mean (SD) excluding rCR	20.6 (11.7)
**Type**	
No special type	47 (87.0%)
Lobular	3 (5.5%)
Spindle cell	1 (1.9%)
Micropapillary	1 (1.9%)
Mucinous	1 (1.9%)
Mixed no special type and mucinous	1 (1.9%)
**Grade**	
1	4 (7.4%)
2	21 (38.9%)
3	29 (53.7%)
ER (Allred score)	
Positive	37 (68.5%)
Negative	17 (31.5%)
PR (Allred score)	
Positive	39 (72.2%)
Negative	15 (27.8%)
Her2	
Positive	4 (7.4%)
Negative	50 (92.6%)

Abbreviations: ER = estrogen receptor; NST = no specific type; rCR = complete radiological response; PR = progesterone receptor; SD = standard deviation.

### Radiotracer administration and timing

Table 2 shows the results and details of the patients administered with ^99m^Tc and ^18^F-FDG. The mean dose of ^18^F-FDG injected was 250.5 MBq (standard deviation, 14.2) and the maximum dose was 273 MBq.

**Table 2.** Patient preparation and surgery.

Patient features (n = 52)	
**^99m^Tc injection**	
Yes	46 (88.5%)
No	6 (11.5%)
Dose of ^99m^Tc (MBq)	
0	6 (11.5%)
20	39 (75.0%)
40	7 (13.5%)
**Dose of ^18^F-FDG**	
210–230	3 (5.8%)
230–250	22 (42.3%)
250–270	24 (46.1%)
270+	3 (5.8%)
Mean (SD)	250.5 (14.2)
Range	213.3–273.0
**Specimen features (n = 54)**	
**Diathermy setting (Watts)**	
Not used	6 (11.1%)
20	31 (57.4%)
25	4 (7.4%)
30	3 (5.6%)
40	8 (14.8%)
45	2 (3.7%)
**Duration of surgery (minutes)**	
<15	13 (24.1%)
15–30	32 (59.3%)
30–60	8 (14.8%)
60+	1 (1.8%)
Mean (SD)	24.4 (19.64)
Range	9–150
Median (IQR)	19.5 (16, 26)
**Time between injection and knife-to-skin (minutes)**	
50–90	14 (25.9%)
90–120	6 (11.1%)
120–180	19 (35.2%)
180–240	12 (22.2%)
240+	3 (5.6%)
Mean (SD)	144.8 (62.5)
Range	50–341
Median (IQR)	138.5 (88–185)

Abbreviations: ^18^F-FDG = ^18^F-fluorodeoxyglucose; ^99m^Tc = ^99m^Tc-albumin-nanocolloid; IQR = interquartile range; SD = standard deviation.

The dose of ^18^F-FDG injected was standardized and checked by the nuclear medicine department. The protocol and pathway created for patients flowed well and allowed them to undergo surgery without any delays to the operating room list. The target dose of 250 MBq (±10%) 18F-FDG was given to 50 of the 52 patients; the remaining 2 received 213.3 MBq and 223.7 MBq, respectively. The reason for lower doses being administered are likely because of the preinjection decay, which was because of timing of delivery. The aim was for the ^18^F-FDG to be administered 145 minutes before imaging.

On average, the first CLI-FAR image was taken 175.1 minutes after the ^18^F-FDG injection; this ranged from 82 to 362 minutes. There was a minimum of 5 minutes between each CLI-FAR image, and most patients had 4 images taken in total.

### Diagnostic performance of CLI-FAR

**Figure 3.**
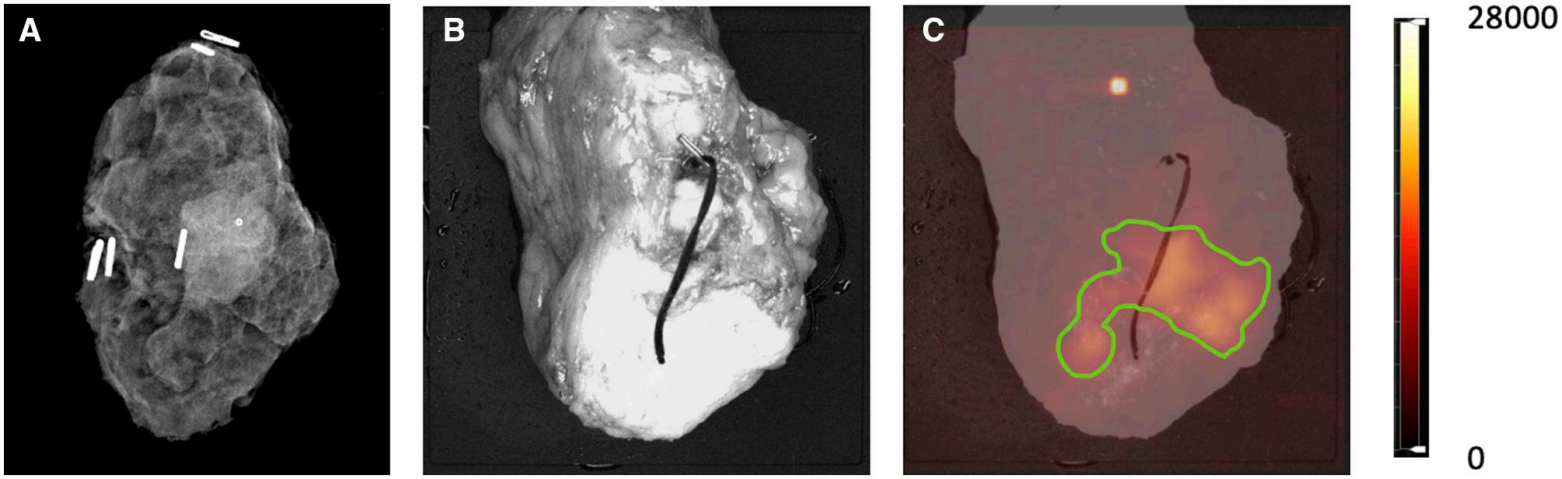
A specimen in which the margins were deemed positive by Cerenkov luminescence imaging (CLI) and negative by intraoperative x-ray. (A) Intraoperative x-ray. (B) White light image. (C) CLI image as seen at the time of interpretation. The green region of interest shows radioactivity on a positive margin. The illuminated spot on the specimen seen above the radioactivity is either contamination or chemiluminescence.

**Figure 4.**
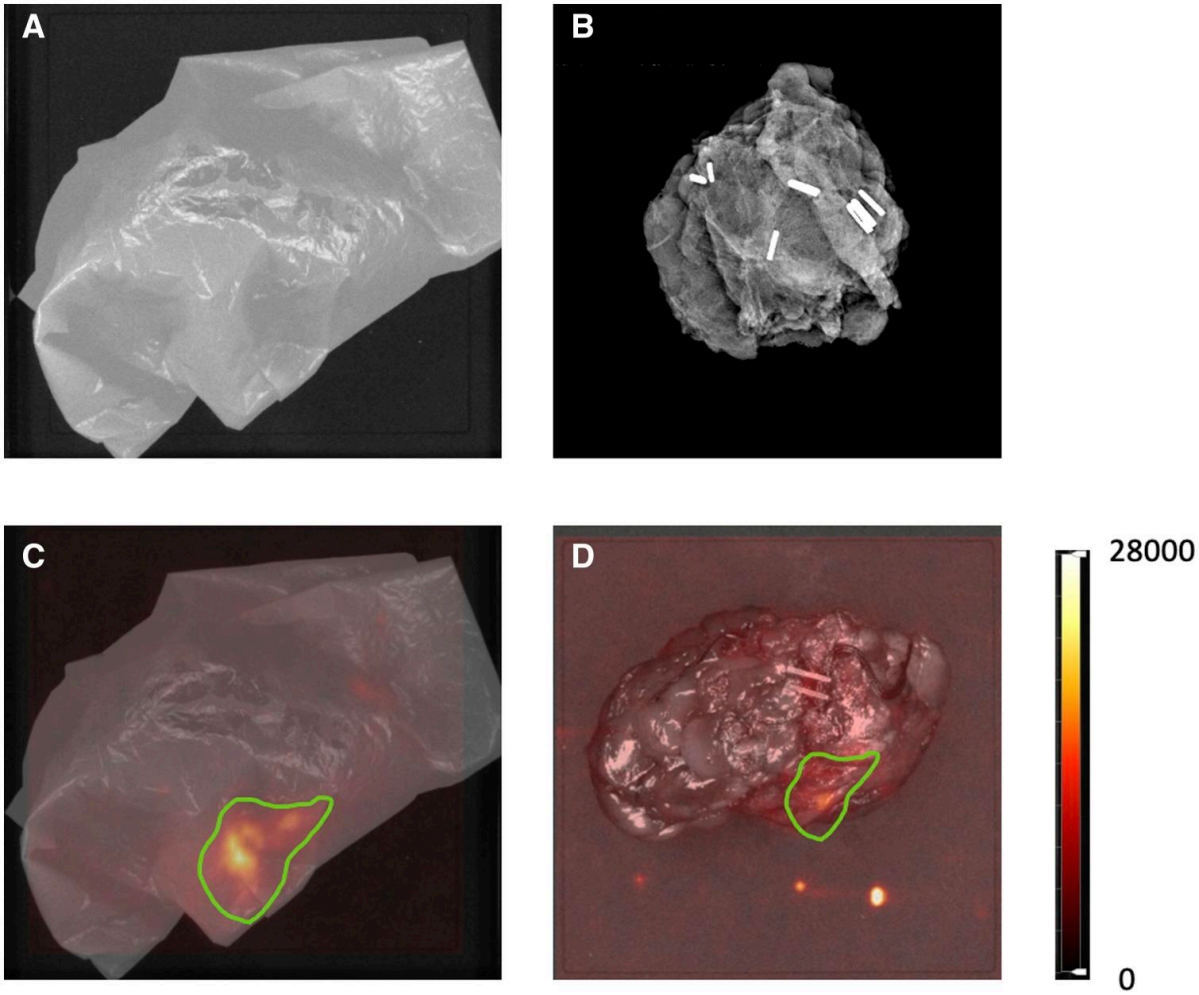
(A) Specimen wrapped in a scintillator, image in black and white. (B) Intraoperative x-ray of the specimen. (C) The flexible autoradiography (FAR) image with a region of interest drawn in green where activity was seen. (D) Cerenkov luminescence imaging (CLI) image of with a region of interest drawn in green where activity was seen.

Of the 54 specimens, 50 had 2 margins assessed with CLI-FAR (results shown in Table S1). The remaining 4 had only 1 margin assessed because of technical errors, leading to a total of 104 margins being assessed with CLI-FAR. Most specimens were assessed using CLI (Figure 3) and FAR imaging (Figure 4); however, 8 specimens were too large for the scintillator, so these were assessed with CLI imaging only. In addition, there was a technical error of system software failure on 1 CLI image; therefore, only 1 margin was assessed using FAR.

In total 13 margins were positive and 91 margins were negative when assessed by final histopathology. CLI-FAR margin assessment was compared with final histopathological assessment (Table 3). This showed a margin specificity of 97.8% (89/91), sensitivity of 76.9% (10/13), positive predictive value of 83.3% (10/12), and negative predictive value of 96.7% (89/92).

**Table 3.** Diagnostic performance of each imaging modality for tumor-positive margins.

104 margins on 54 specimens in 52 patients (95% CI)				
	X-ray	CLI	FAR	CLI-FAR
Sensitivity	(2/13) 15.4%	(10/13) 76.9%	(3/9) 33.3%	(10/13) 76.9%
	(8.5–22.3)	(68.8–85.1)	(23.4–43.3)	(68.3–85.0)
Specificity	(82/91) 90.1%	(86/90) 95.6%	(76/77) 98.7%	(89/91) 97.8%
	(84.4–95.9)	(91.6–99.5)	(96.3–101.1)	(95.0–100.6)
PPV	(2/11) 18.2%	(10/14) 71.4%	(3/4) 75.0%	(10/12) 83.3%
	(10.8–25.6)	(92.47–80.2)	(65.9–84.2)	(76.2–90.5)
NPV	(82/93) 88.2%	(86/89) 96.6%	(76/82) 92.7%	(89/92) 96.7%
	(82.0–94.1)	(93.1–100.1)	(65.9–84.2)	(93.3–100.2)
Number of margins assessed per specimen				
1		4 (7.4%)		
2		50 (92.6%)		
Number of margins with lack of concordance (CLI-FAR vs histopathology)				
0		49 (90.7%)		
1		5 (9.3%)		

Abbreviations: CI = confidence interval; CLI = Cerenkov luminescence imaging; FAR = flexible autoradiography; NPV = negative predictive value; PPV = positive predictive value.

The diathermy setting used for 1 specimen imaged with CLI only was relatively high at 30 W and the false-positive result can be attributed to chemiluminescence. Furthermore, the time between ^18^F-FDG injection and CLI imaging for 2 false-positive interpretations were 97 and 99 minutes, significantly lower than the planned 145 minutes, which could have contributed to this result.

### Reexcision rate

In all, 10 margins in 8 patients were correctly identified as positive on CLI-FAR imaging, which were acted on intraoperatively. In these patients, all initial margins were also positive on histopathology, but cavity shavings were benign on 7; therefore, these patients avoided a second operation. One patient with a positively identified margin on CLI-FAR underwent an intraoperative cavity shave, and the excised cavity shave on histopathology showed further disease at the new margin. The histopathology report showed 3 false-negative interpretations of margins on CLI-FAR. One specimen had a positive margin for DCIS. The other 2 specimens had invasive cancer at the margin. The reexcision rate using CLI-FAR was 7.7% (4/52). The overall reexcision rate was decreased by 69% (17.3/25).

Of 52 patients, 3 patients’ margins assessed as negative on CLI-FAR required further surgery because of invasive cancer or associated DCIS within 1 mm of the margin. Of the 3 specimens that were incorrectly considered to be negative, 2 were too large to be assessed with FAR and were only assessed using CLI imaging.

### Comparison with conventional x-ray

CLI-FAR identified positive and negative margins more frequently than intraoperative x-ray. Intraoperative x-ray correctly identified 82 of the 91 negative margins on histopathology, but incorrectly identified 9 as positive, leading to more healthy tissue being excised in the form of cavity shavings. Intraoperative x-ray correctly identified 2 of the 13 positive margins on histopathology, whereas CLI-FAR identified 10 margins correctly.

### Surgical time

The addition of CLI-FAR for imaging did not significantly prolong surgical time (Table 4). There was significant variation in the time between administration of ^18^F-FDG and the start of surgery, and the duration of surgery, leading to a wide range of time between ^18^F-FDG injection and procuring CLI-FAR images. These time delays are summarized in Table 3. For most patients, the delay between surgical resection and CLI-FAR imaging was minimal (a mean of 6 minutes).

**Table 4.** Time between key points of the intraoperative specimen analysis procedure.

Specimen specific (n = 54)	
**Time between injection and intraoperative x-ray (minutes)**	
60–90	3 (5.5%)
90–120	11 (20.4%)
120–180	19 (35.2%)
180–240	14 (25.9%)
240+	7 (13.0%)
Mean (SD)	169.1 (64.6)
Range	74–356
Median (IQR)	163 (113–217)
**Time between tracer injection and first CLI-FAR image (minutes)**	
60–120	13 (24.1%)
120–180	20 (37.0%)
180–240	13 (24.1%)
240+	8 (14.8%)
Mean (SD)	175.1 (63.2)
Range	82–362
Median (IQR)	167.5 (131–221)
**Time between intraoperative x-ray and first CLI-FAR image (minutes)**	
<3	6 (11.1%)
3–4	16 (29.6%)
5	11 (20.4%)
6–7	13 (24.1%)
>7	8 (14.8%)
Mean (SD)	6.0 (5.6)
Range	0–40
Median (IQR)	5 (4–6)
**Time between first image and last image (minutes)**	
<11	9 (16.7%)
11–15	25 (46.3%)
16–20	9 (16.7%)
>20	11 (20.32%)
Mean (SD)	15.4 (5.6)
Range	6–31
Median (IQR)	14 (13–20)

Abbreviations: CLI-FAR = Cerenkov luminescence imaging–flexible autoradiography; IQR = interquartile range; SD = standard deviation.

## Discussion

This first-in-human study evaluated BCS specimen margin assessment using ^18^F-FDG for CLI-FAR imaging. In all, 104 margins were assessed in 54 specimens; 46 specimens underwent both imaging modalities of CLI and FAR. Overall, 8 specimens were too large for the scintillator to cover the specimen, hence FAR was not undertaken. Margin correlation was good between CLI-FAR imaging and final histopathology with 7 of 8 patients avoiding a second operation because of positive margins as detected by CLI-FAR. CLI-FAR shows a margin specificity of 97.8% (89/91) and a sensitivity of 76.9% (10/13). The reexcision rate using CLI-FAR was 7.7% (4/52), which is lower than the current reported 20% to 25% for intraoperative x-ray.^2–5^

CLI-FAR's dual high-resolution imaging technique uses imaging equipment that can be used in the operating room. CLI-FAR is particularly useful for image-guided surgery with an acquisition time of 10 minutes per margin and instant image analysis. Grootendorst et al. showed proof of principle using CLI on 10 excised specimens observing radioactivity in tumor cells and followed this with assessment of 15 margins in 12 patients,^16^ in which all margins were negative for invasive cancer on imaging and histopathology. Jurrius et al. investigated 385 margins on BCS specimens in 66 patients using FAR in a multicenter trial in Poland, and showed 46.2% sensitivity, 81.7% specificity, 8.1% positive predictive value, 97.7% negative predictive value, and overall accuracy of 80.5%, detecting both invasive carcinoma and DCIS.^17^ These studies have previously shown that radiation exposure is low and safe for staff members^16^^,^^22^; this has also been reflected in other ^18^F-FDG breast assessment studies.^24^^,^^25^

A total of 5 surgical consultants were involved in the study, from recruitment to assessing the CLI-FAR images. Thus, different interpreters were able to use the technology.

### Future work

The current study has already identified that DCIS associated with invasive cancer is detected on CLI-FAR imaging assessment. However, it is unknown whether pure DCIS can be identified using CLI-FAR imaging. Therefore, the CLI-FAR study is to be extended to include a further cohort of patients with pure DCIS requiring BCS.

## Limitations

Both CLI and FAR have limitations in assessing positive margins. Chemiluminescence continues to be a limiting factor in CLI, which requires teaching and experience to successfully differentiate it from radioactivity. In this study, a standard scintillator that could only accommodate specimens up to 4 cm in size was used. However, given the growing prevalence of oncoplastic surgical procedures, it is becoming more frequent for specimens to exceed 4 cm in diameter. For such specimens, larger scintillators are now readily available. Because this is a feasibility study representing a relatively small series of patients, a larger validation series may be required for confirmation in the future.^26–31^

## Conclusion

CLI-FAR assessment of margins in BCS can be feasibly integrated into standard clinical care. It enables surgeons to accurately assess margin status specimens intraoperatively when compared with gold standard histopathologic examination.

## Supplementary Material

umae015_Supplementary_Data

## Data Availability

Data underlying this article will be shared on reasonable request to the corresponding author.

## Abbreviations

^18^F-FDG: ^18^F-fluorodeoxyglucose
^99^mTc: ^99^mTc-albumin-nanocolloid
BCS: breast-conserving surgery
CLI-FAR: Cerenkov luminescence imaging–flexible autoradiography
DCIS: ductal carcinoma in situ
NACT: neoadjuvant chemotherapy
