# Supplementary material for: Cerenkov luminescence imaging and flexible autoradiography for specimen margin assessment during breast-conserving cancer surgery
Source: Radiol Adv. 2024 May 24;1(2):umae015. doi: 10.1093/radadv/umae015 (PMC12483239; doi:10.1093/radadv/umae015)
Supplement: umae015_Supplementary_Data [file umae015_Supplementary_Data.zip › Supplemental Materials 19052024.docx]

**Cerenkov Luminescence Imaging and Flexible Autoradiography for specimen margin assessment during breast-conserving cancer surgery**

Aaditya Sinha^1,2^, Zhane Peterson^1,2^, Belul Shifa^1,2^, Hannah Jeffery^1,2^, Patriek Jurrius^1,2^, Sarah Allen^1,2^, Eugene Lee^2^, Mohammed Azmat^2^, Rachel Barrass^2^, Damion Bailey^2^, Jessica Johnson^2^, Kathryn Adamson^2^, Vasileios Karydakis^2^, Elina Shaari^2^, Mangesh Thorat^2^, Hisham Hamed^2^, Georgina Bitsakou^2,^ Sarah Pinder^1,2^, Padma Menon^2^, Wen Ng^2^, Gary Cook^1,2^, John Joemon ^2^, Armidita Jacob^2^, Sofia Pereira^2^, Jocelyn Thomas^2^, Ruheana Begum^2^, Karim El-Boghdadly^1,2^, Mieke Van Hemelrijck^1,2^, Ashutosh Kothari^1,2^, Arnie Purushotham^1,2^

^1^King’s College London

^2^Guy's and St Thomas’ NHS Foundation Trust

Corresponding Author: Professor Arnie Purushotham

Email Address: [arnie.purushotham@kcl.ac.uk](mailto:arnie.purushotham@kcl.ac.uk)

Mailing Address: 3^rd^ Floor Bermondsey Wing, Guy’s Hospital, Great Maze Pond, London, SE1 9RT

**Supplemental Figures Captions**

| **Figure** |  |
| --- | --- |
|  |  |
|  |  |
| **S1** | *This figure shows the pathway that was followed for patients to be informed of the study and them providing their consent to participate in the study. There were multiple steps to this and this has been shown in this figure. CLI-FAR patient consent pathway, Abbreviations: MDM – Multi-disciplinary Meeting, OPA – Outpatient Appointment, EPR – Electronic Patient Records, CTC – Clinical Trial Coordinator, CNS – Cancer Nurse Specialist, SPAR – Surgical Preparation and Recovery.* |
| **S2** | *This figure shows the pathway for organising and ensuring all team members were aware of patients that had provided their consent to participate in the study and when their procedure was taking place. There were multiple steps to this which has been shown in this figure. Pathway to ensure that patients consented to CLI-FAR are identified in advance and are placed on the theatre list, ensuring patients not participating are not incorrectly tagged as CLI-FAR Abbreviations: TCI – To Come In, CTC – Clinical Trial Coordinator, CNS – Cancer Nurse Specialist, SPAR - Surgical Preparation and Recovery.* |
| **S3** | *This figure shows a checklist that required to be completed for all patients prior to them having an injection of ^18^F-FDG on the day of surgery.* |
| **S4** | *This figure shows the pathway followed on the day of surgery from the point the participating patient attended the hospital for their surgery till they were discharged and what steps were completed.* |
| **S5** | *This figure shows that there is a difference in the degree of diathermy artifact when a higher energy level is used. It further shows how the luminescence of the heat energy decreases over time as the area exposed to the diathermy cools down. The image compares three different diathermy settings. A. Diathermy artifact on pre-clinical studies, time shown on the x-axis is in minutes, Diathermy levels are in Watts and the numbers below the images expresses the maximum brightness in luminescence units. B. Diathermy artifact on pre-clinical studies, depicted in a linear-like relationship graph.* |

**Supplemental Table Captions**

| **Table** |  |
| --- | --- |
| **S1** | Intraoperative margin assessment methods with their corresponding status in clinical practice. |
| **S2** | Demographics of each patient |

**Supplemental Figures**

 ***Figure S1 -*** *This figure shows the pathway that was followed for patients to be informed of the study and them providing their consent to participate in the study. There were multiple steps to this and this has been shown in this figure. CLI-FAR patient consent pathway, Abbreviations: MDM – Multi-disciplinary Meeting, OPA – Outpatient Appointment, EPR – Electronic Patient Records, CTC – Clinical Trial Coordinator, CNS – Cancer Nurse Specialist, SPAR – Surgical Preparation and Recovery.*

** *Figure S2 –*** *This figure shows the pathway for organising and ensuring all team members were aware of patients that had provided their consent to participate in the study and when their procedure was taking place. There were multiple steps to this which has been shown in this figure. Pathway to ensure that patients consented to CLI-FAR are identified in advance and are placed on the theatre list, ensuring patients not participating are not incorrectly tagged as CLI-FAR Abbreviations: TCI – To Come In, CTC – Clinical Trial Coordinator, CNS – Cancer Nurse Specialist, SPAR - Surgical Preparation and Recovery.*

**

***Figure S3****- This figure shows a checklist that required to be completed for all patients prior to them having an injection of ^18^F-FDG on the day of surgery.*

***Figure S4 -****This figure shows the pathway followed on the day of surgery from the point the participating patient attended the hospital for their surgery till they were discharged and what steps were completed.*


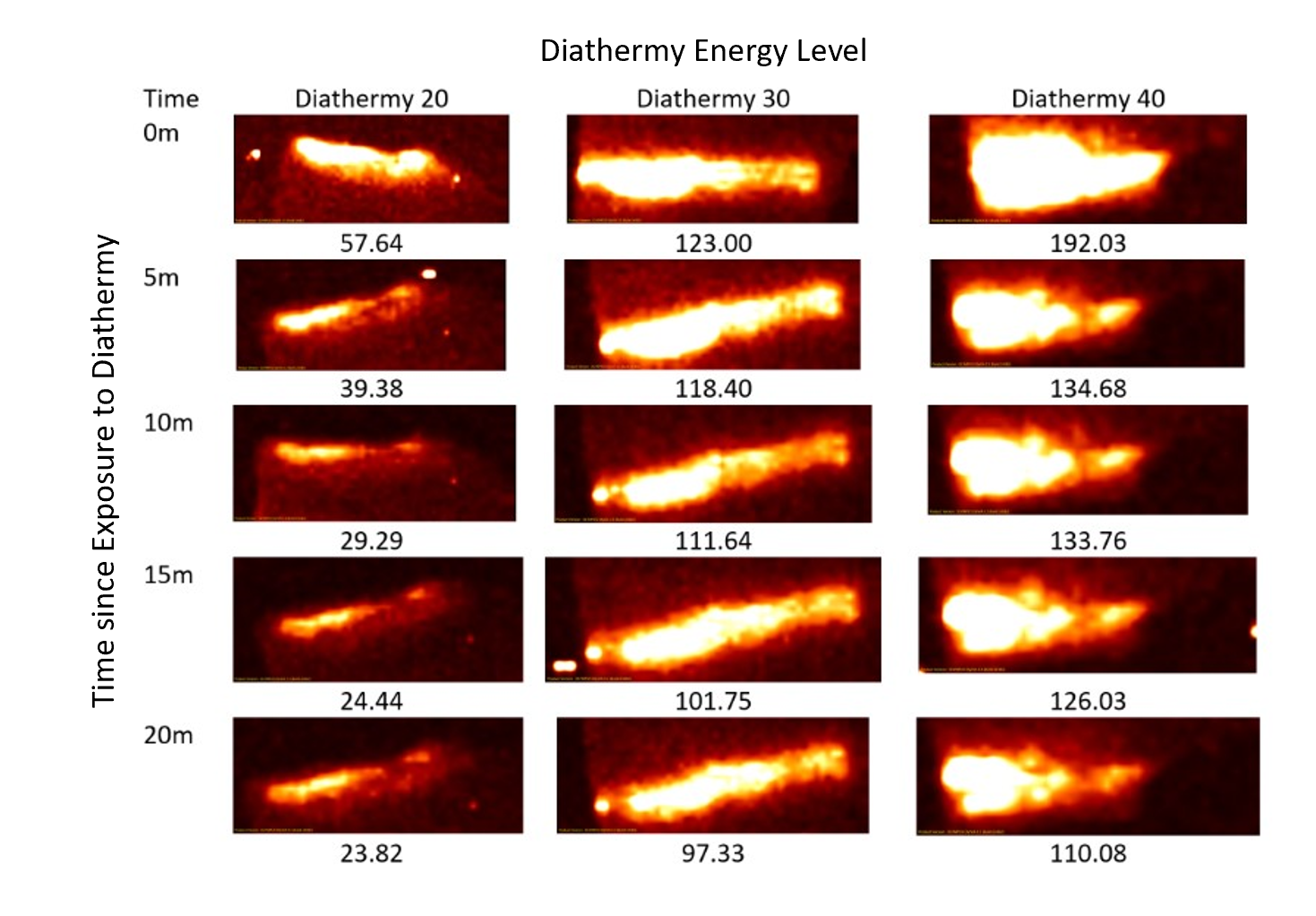

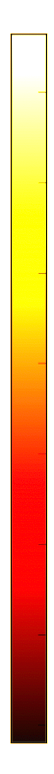


A

200

0


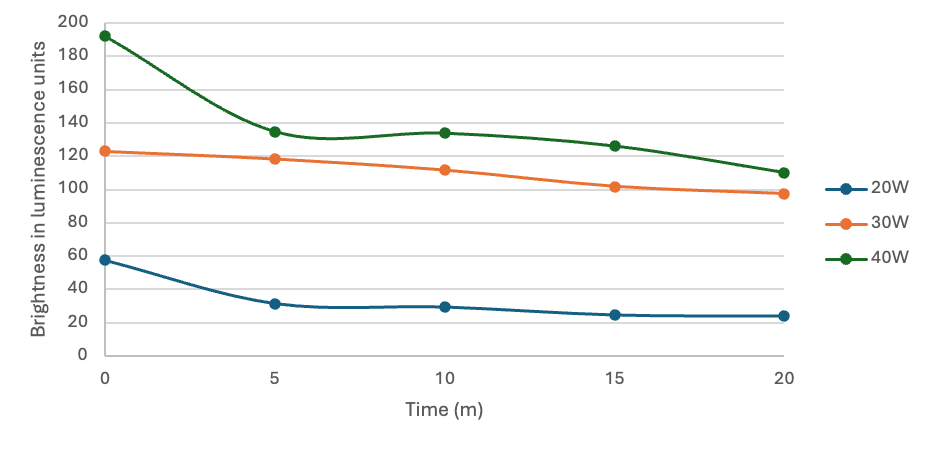


B

***Figure S5*** *– This figure shows that there is a difference in the degree of diathermy artifact when a higher energy level is used. It further shows how the luminescence of the heat energy decreases over time as the area exposed to the diathermy cools down. The image compares three different diathermy settings. A. Diathermy artifact on pre-clinical studies, time shown on the x-axis is in minutes, Diathermy levels are in Watts and the numbers below the images expresses the maximum brightness in luminescence units. B. Diathermy artifact on pre-clinical studies, depicted in a linear-like relationship graph.*

**Supplementary Tables**

***Table S1 – Intraoperative margin assessment methods with their corresponding status in clinical practice.***

| Intra-operative Imaging Modality | Current Status |
| --- | --- |
| Frozen Section | Clinically used |
| Ultrasound | Clinically used |
| Intraoperative X-Ray | Clinically used |
| Radiofrequency spectroscopy | Clinically used |
| Enzyme-activable fluoroscopy | Experimental |
| Protein-targeting Fluoroscopy | Experimental |
| In-cell based fluorescent Probe | Experimental |
| Magnetic Resonance Imaging | Experimental |
| Raman Spectroscopy | Experimental |
| Ambient Mass Spectrometry | Experimental |
| Optical Coherence Tomography | Experimental |
| Diffuse Spectroscopy | Experimental |
| Confocal Microscopy | Experimental |
| Molecular Imaging (e.g. Positron Emission Tomography) | Experimental |

***Table S2: Demographics of each patient***

| **Patient** | **Tumour** | **Age [years]** | **Ethnicity** | **Tumour side** | **Tumour size [mm]** | **Tumour type** | **Tumour grade** | **ER [Allred]** | **PR [Allred]** | **Her-2 [Allred/FISH]** | **NACT** | **RCB** |
| --- | --- | --- | --- | --- | --- | --- | --- | --- | --- | --- | --- | --- |
| 1 | 1 | 42 | White | Left | 14 | NST | 3 | 7 | 8 | Negative | No |  |
| 2 | 1 | 51 | White | Left | 32 | NST | 3 | 4 | 4 | Negative | Yes | 2 |
| 3 | 1 | 50 | White | Right | 16 | NST | 2 | 7 | 7 | Negative | No |  |
| 4 | 1 | 71 | White | Right | 45 | NST | 2 | 8 | 8 | Negative | No |  |
| 5 | 1 | 61 | Black | Left | 41 | Spindle Cell | 3 | 0 | 0 | Negative | No |  |
| 6 | 1 | 47 | Black | Right | 30 | NST | 2 | 7 | 6 | Positive | Yes | 2 |
| 7 | 1 | 47 | Other | Right | 18 | Mixed NST and mucinous | 3 | 7 | 6 | Negative | No |  |
| 8 | 1 | 51 | White | Left | 12.5 | NST | 3 | 0 | 4 | Positive | Yes | 0 |
| 9 | 1 | 46 | Black | Right | 19 | NST | 3 | 0 | 0 | Negative | Yes | 0 |
| 9 | 2 | 46 | Black | Left | 10 | NST | 3 | 0 | 0 | Negative | Yes | 0 |
| 10 | 1 | 52 | White | Right | 41 | NST | 3 | 8 | 4 | Negative | No |  |
| 11 | 1 | 65 | White | Right | 4 | NST | 1 | 7 | 5 | Negative | No |  |
| 12 | 1 | 52 | White | Left | 21 | Lobular | 2 | 8 | 8 | Negative | Yes | Not reported |
| 13 | 1 | 46 | Black | Right | 20 | NST | 3 | 0 | 0 | Negative | Yes | 0 |
| 14 | 1 | 62 | White | Right | 10 | NST | 3 | 7 | 8 | Negative | No |  |
| 15 | 1 | 46 | White | Left | 0 | NST | 3 | 0 | 0 | Negative | Yes | 0 |
| 16 | 1 | 55 | White | Right | 10 | NST | 1 | 7 | 8 | Negative | No |  |
| 17 | 1 | 58 | White | Right | 9 | NST | 3 | 0 | 0 | Negative | Yes | 0 |
| 18 | 1 | 59 | White | Left | 7 | Lobular | 2 | 8 | 8 | Negative | No |  |
| 18 | 2 | 59 | White | Left | 11 | NST | 2 | 8 | 8 | Negative | No |  |
| 19 | 1 | 42 | Asian | Right | 0 | NST | 3 | 7 | 8 | Positive | Yes | 0 |
| 20 | 1 | 58 | White | Left | 16 | NST | 2 | 8 | 7 | Negative | No |  |
| 21 | 1 | 48 | White | Left | 20 | NST | 3 | 6 | 8 | Negative | Yes | 2 |
| 22 | 1 | 52 | White | Right | 6.5 | NST | 2 | 7 | 8 | Negative | No |  |
| 23 | 1 | 48 | White | Right | 56 | NST | 3 | 6 | 6 | Negative | No |  |
| 24 | 1 | 50 | White | Left | 28 | NST | 2 | 8 | 8 | Negative | No |  |
| 25 | 1 | 62 | White | Left | 21 | NST | 2 | 8 | 7 | Negative | No |  |
| 26 | 1 | 39 | White | Right | 0 | NST | 3 | 0 | 0 | Negative | Yes | 0 |
| 27 | 1 | 72 | Chinese | Left | 24 | NST | 3 | 8 | 8 | Negative | No |  |
| 28 | 1 | 49 | Black | Left | 31 | NST | 3 | 8 | 7 | Negative | No |  |
| 29 | 1 | 56 | White | Left | 8 | Micropapillary | 2 | 8 | 8 | Negative | No |  |
| 30 | 1 | 63 | Black | Left | 28 | Lobular | 2 | 7 | 0 | Negative | No |  |
| 31 | 1 | 84 | White | Left | 25 | NST | 2 | 8 | 8 | Negative | No |  |
| 32 | 1 | 31 | Black | Left | 0 | NST | 3 | 0 | 2 | Negative | Yes | 0 |
| 33 | 1 | 22 | Black | Left | 0 | NST | 3 | 0 | 0 | Negative | Yes | 0 |
| 34 | 1 | 43 | White | Right | 0 | NST | 3 | 0 | 0 | Positive | Yes | 0 |
| 35 | 1 | 67 | White | Left | 0 | NST | 2 | 8 | 4 | Negative | Yes | 0 |
| 36 | 1 | 62 | White | Left | 13 | NST | 2 | 8 | 7 | Negative | No |  |
| 37 | 1 | 59 | Black | Left | 0 | NST | 3 | 0 | 0 | Negative | Yes | 0 |
| 38 | 1 | 44 | White | Right | 0 | NST | 3 | 0 | 0 | Negative | Yes | 0 |
| 39 | 1 | 41 | Black | Left | 0 | NST | 3 | 0 | 4 | Negative | Yes | Not assessed |
| 40 | 1 | 48 | Other | Left | 0 | NST | 2 | 0 | 0 | Negative | Yes | 0 |
| 41 | 1 | 56 | Black-Caribbean | Right | 28 | NST | 2 | 8 | 7 | Negative | No |  |
| 42 | 1 | 80 | White | Right | 32 | NST | 3 | 8 | 5 | Negative | No |  |
| 43 | 1 | 73 | White | Left | 37 | NST | 3 | 8 | 8 | Negative | No |  |
| 44 | 1 | 49 | Not Specified | Left | 17 | NST | 2 | 8 | 8 | Negative | No |  |
| 45 | 1 | 62 | White | Left | 18 | NST | 2 | 8 | 8 | Negative | No |  |
| 46 | 1 | 47 | White | Right | 10 | NST | 3 | 3 | 4 | Negative | No |  |
| 47 | 1 | 77 | Black-Caribbean | Right | 17 | NST | 3 | 0 | 0 | Negative | No |  |
| 48 | 1 | 66 | Black-Caribbean | Right | 8 | NST | 2 | 8 | 8 | Negative | No |  |
| 49 | 1 | 40 | Not Specified | Left | 10 | NST | 3 | 7 | 8 | Negative | Yes | 0 |
| 50 | 1 | 59 | White | Left | 13 | NST | 1 | 8 | 8 | Negative | No |  |
| 51 | 1 | 78 | White | Left | 23 | Mucinous | 1 | 8 | 8 | Negative | No |  |
| 52 | 1 | 72 | White | Right | 25 | NST | 2 | 8 | 8 | Negative | No |  |
