# Supplementary material for: Cerenkov luminescence imaging and flexible autoradiography for specimen margin assessment during breast-conserving cancer surgery
Source: Radiol Adv. 2024 May 24;1(2):umae015. doi: 10.1093/radadv/umae015 (PMC12483239; doi:10.1093/radadv/umae015)
Supplement: umae015_Supplementary_Data [file umae015_Supplementary_Data.zip › 20220815 CLI-FAR Protocol v1.1signed.pdf]

CLI-FAR

Intraoperative assessment of tumour excision margins using the LightPath® Imaging System for Cerenkov luminescence imaging (CLI) combined with flexible autoradiography (FAR) in women undergoing breast-conserving surgery (BCS)

Short title: *CLI and FAR for Intraoperative Margin Assessment*

REC reference: 15/LO/0029
IRAS number: 314460
Clinicaltrials.gov NCT05496101

Version: 1.1, 15 August 2022 SUPERSEDES: - V1. 17 June 2022

Sponsor: King's College London
&
Guy's & St. Thomas' NHS Foundation Trust

CONFIDENTIALITY STATEMENT

The information in this document contains trade secrets and commercial information that are privileged or confidential and may not be disclosed unless such disclosure is required by applicable law or regulations. In any event, persons to whom the information is disclosed must be informed that the information is privileged or

KING'S HEALTH PARTNERS
An Academic Health Sciences Centre for London
Pioneering better health for all

confidential and may not be further disclosed by them. These restrictions on disclosure will apply equally to all future information supplied to you which is indicated as privileged or confidential.

Intraoperative assessment of tumour excision margins using the LightPath® Imaging System for Cerenkov luminescence imaging (CLI) combined with flexible autoradiography (FAR) in women undergoing breast-conserving surgery (BCS)

Clinical Study Reference: CLI-FAR

Protocol Version: 1.1, 15 August 2022

Protocol Approval Page

The undersigned have read this Study Protocol and hereby confirm that, to the best of their knowledge, it accurately describes the clinical study to be conducted:

Chief Investigator

Professor Arnie Purushotham<br>Kings College London	______________________<br>Signature	15 August 2022<br>______________<br>Date

KING'S HEALTH PARTNERS
An Academic Health Sciences Centre for London
Pioneering better health for all

Intraoperative assessment of tumour excision margins using the LightPath® Imaging System for Cerenkov luminescence imaging (CLI) combined with flexible autoradiography (FAR) in women undergoing breast-conserving surgery (BCS)

Clinical Study Reference: CLI-FAR

Protocol Version: 1.1, 15 August 2022

Investigator Agreement:

I have read this protocol and agree to conduct the study as outlined herein, and according to Good Clinical Practice E6 (R2).

I will provide copies of the protocol and all pertinent information to all individuals responsible to me who assist in the conduct of this study. I will discuss this material with them to ensure they are fully informed regarding the investigational device and the conduct of the study.

Prof. Arnie Purushotham

15 August 2022

Investigator Signature ______________________ Date ______________

Mr Ashutosh Kothari

Investigator Signature ______________________ Date ______________

Contents

1. Investigators ... 8
1.1. Principal investigators ... 8
1.2. Co-investigators ... 8
1.3. Participating study site ... 8
2. General Information ... 9
2.1. Abbreviations ... 9
2.2. Synonyms ... 11
2.3. Synopsis ... 11
2.4. Schedule of Events ... 16
3. Background Information ... 17
3.1. Wide Local Excision for Breast Cancer ... 17
3.2. LightPath® Imaging ... 18
3.2.1. Cerenkov Luminescence Imaging (CLI) ... 18
3.2.2. Flexible Autoradiography (FAR) ... 18
3.2.3. Benefits ... 18
4. Administration of Radiopharmaceuticals ... 19
4.1. Administration of ^{18}F-fluorodeoxyglucose (^{18}F-FDG) ... 19
4.1.1. Rationale ... 19
4.1.2. Dose ... 19
4.1.3. Regulatory Status ... 19
4.1.4. Additional Information ... 19
4.2. Administration of Technetium 99m (^{99m}Tc tracer) ... 19
4.2.1. Rationale ... 19
4.2.2. Dose ... 19
4.2.3. Regulatory Status ... 19
4.2.4. Licence Requirements ... 20
4.2.5. Radiation Safety ... 20
4.2.6. Handling of Radioactive Materials ... 20
5. Imaging device ... 20
5.1. Imaging system ... 20
5.2. Regulatory Status of Product ... 20
5.3. Description of Device ... 20
5.4. Packaging and Labelling of Device ... 21
5.5. Device Training ... 21
6. Study Objectives ... 22
6.1. Primary outcome measure ... 22
6.2. Secondary outcome measures ... 22
7. Study Design ... 22
7.1. Overview of Study Design ... 22
7.2. Risks and Benefits ... 22
7.2.1. Risks associated with LightPath® directed surgical procedures ... 22
7.2.2. Risk to participants ... 23
7.2.3. Radiation exposure associated risks from ^{18}F-FDG ... 23

7.2.4. Radiation exposure associated risks from ^{99m}Tc-nanocolloid 23
7.2.5. Combined radiation exposure associated risk of ^{18}F-FDG and ^{99m}Tc 24
7.2.6. Risk to staff 24
7.2.7. Benefits to Participants 26

7.3. Study Population 26
7.4. Inclusion Criteria 26
7.5. Exclusion Criteria 27

8. Study Procedures 27
8.1. Study Start and Training 27
8.2. Recruitment 27
8.3. Informed Consent 27
8.4. Baseline Data 28
8.5. Concomitant Medication/Treatments 28
8.6. Day of surgery 28
8.6.1. Radiopharmaceutical administration 28
8.6.2. BCS only 29
8.6.3. BCS plus axillary surgery (SLNB or ALND) 29
8.6.4. LightPath® assessment 29
8.6.5. Imaging of the WLE specimen 29
8.6.6. Imaging of the cavity shavings 30
8.6.7. Radiation safety 30
8.6.8. Histopathological assessment 30
8.6.9. Safety 30

8.7. Follow-up 30
8.8. Study Duration 31
8.8.1. Duration of Subject Participation 31
8.8.2. Duration of the Clinical Study 31

8.9. Withdrawal of Subjects 31
8.10. Handling of Radioactive Materials 31

9. Study Monitoring and Auditing 31

10. Adverse Events Definition and Reporting 31
10.1. Adverse Event Definition 31
10.2. Serious Adverse Event Definition 32
10.3. Anticipated Adverse Events 32
10.4. Adverse Event Severity 33
10.5. Relationship to Study Procedures 33
10.6. Adverse Event Reporting 34
10.7. Serious Adverse Event Reporting 35
10.8. Device Deficiencies 35

11. Data Collection, Processing and Statistical Analysis 36
11.1. Completion of Data Collection Forms 36
11.2. Data Correction 36
11.3. Review and Return of Completed Documentation 36
11.4. Data Protection 36
11.5. Record Retention 37

11.6. Data Analysis 37
11.6.1. Analysed Population 37
11.6.2. Sample Size Determination 38
11.7. Data Analysis for Outcome Measures 38
11.7.1. Analysis of the Primary Outcome Measure 38
11.7.2. Analysis of Secondary Outcome Measures 39
11.7.3. Safety Analysis 40
11.8. Termination / Drop-out 40
12. Statement of Compliance 40
12.1. Insurance 41
13. Quality Control and Quality Assurance 41
14. Protocol Amendments 41
14.1. Procedure for Reporting Deviations from the Protocol 41
15. Device Accountability and Storage 41
16. References ……….. 42

1. Investigators

1.1. Principal investigators

**Prof. Arnie Purushotham**	Professor of Breast Cancer – King's College London (KCL)<br>Consultant Surgeon – Guy's and St Thomas (GSTT)
**Mr Ashutosh Kothari**	Clinical lead Breast Surgery – GSTT

1.2. Co-investigators

**Prof. Sarah Pinder**	Professor of Breast Pathology – KCL<br>Consultant Breast Pathologist – GSTT
**Prof. Gary Cook**	Professor of PET imaging – KCL<br>Honorary Consultant Physician PET imaging – GSTT
**Dr Sarah Allen**	Head of Nuclear Medicine – GSTT
**Mr Hisham Hamed**	Consultant Breast Surgeon – GSTT
**Ms Georgina Bitsakou**	Consultant Breast Surgeon – GSTT
**Dr Cheryl Gillett**	Head of the KHP Biobank – KCL
**Ms Ruheana Begum**	Matron of Guy's Tower Theatres – GSTT
**Mr Aaditya Sinha**	MD(Res) student – KCL
**Dr Patriek Jurrius**	PhD student – KCL<br>Honorary Clinical Fellow Breast Surgery – GSTT

1.3. Participating study site

Guy's Hospital
Great Maze Pond
London
SE1 9RT
United Kingdom

2. General Information

2.1. Abbreviations

^{18}F-FDG	^{18}F-fluorodeoxyglucose
^{99m}Tc	Technetium-99m nannocolloid
AE	Adverse Event
ALARP	As low as reasonably practicable
ALND	Axillary Lymph Node Dissection
BCS	Breast-conserving Surgery
cALND	completion Axillary Lymph Node Dissection
CLI	Cerenkov Luminescence Imaging
CRF	Case Report Form
CT	Computed Tomography
CTCAE	Common Terminology Criteria for Adverse Events
DCF	Data Clarification Form
DCIS	Ductal Carcinoma *In Situ*
emCCD	electron multiplying Charge Coupled Device
ER	Oestrogen Receptor
FAR	Flexible AutoRadiography
GCP	Good Clinical Practice
GSTT	Guy's and St. Thomas' NHS Foundation Trust
HER2	Human epidermal-growth-factor receptor 2
IB	Investigator's Brochure
ICH	International Conference on Harmonization
ICRP	International Commission on Radiological Protection
IFU	Instructions for Use
ITT	Intention to treat
IV	Intravenous
KCL	King's College London
MDT	Multidisciplinary team
PET	Positron Emission Tomography
PI	Principal Investigator

PIS	Patient Information Sheet
PR	Progesterone receptor
SAE	Serious Adverse Event
SLN	Sentinel Lymph Node
SLNB	Sentinel Lymph Node Biopsy
WL	White Light
WLE	Wide Local Excision

2.2. Synonyms

The following terms are considered to be synonymous:

Study Trial

2.3. Synopsis

**Study Title**	Intraoperative assessment of tumour excision margins using the LightPath® Imaging System for Cerenkov luminescence imaging (CLI) combined with flexible autoradiography (FAR) in women undergoing breast-conserving surgery (BCS)
**Short Study Title**	CLI and FAR for Intraoperative Margin Assessment
**Study Reference**	*CLI-FAR*
**Intervention**	Intraoperative CLI + FAR LightPath® imaging
**Comparator**	Standard-of-care histopathology (gold standard)
**Product Regulatory Status and Study Phase**	Imaging System:<br>The LightPath® Imaging System is an *in vitro* diagnostic device which has CE mark in Europe, in line with *In Vitro* Diagnostic Medical Devices 98/79/EC (post-marketing study).<br><br>Radiopharmaceutical:<br>^{18}F-FDG is a routinely used Positron Emission Tomography (PET) / Computed Tomography (CT) radiopharmaceutical<br><br>Study phase:<br>Phase II Study
**Study Sponsors**	King's College London<br>Guy's and St. Thomas' NHS Foundation Trust
**Study Site**	**Guy's Hospital**<br>Great Maze Pond<br>London<br>SE1 9RT<br>United Kingdom

**Clinical indication**	Breast-conserving surgery by means of a wide-local excision (WLE) for breast cancer. WLE is also known as a lumpectomy.
**Study Population**	Women ≥18 years of age with invasive breast cancer scheduled for primary breast-conserving surgery with or without sentinel lymph node biopsy (SLNB) or axillary lymph node dissection (ALND)
**Study Design**	Interventional, Open, Single Arm
**Purpose**	This study is a prospective, single arm interventional study to evaluate the diagnostic accuracy of intraoperative Cerenkov luminescence imaging (CLI) plus flexible autoradiography (FAR) using the LightPath® Imaging System for intraoperative tumour margin assessment compared to post-operative standard-of-care histopathology in women undergoing breast-conserving surgery for breast cancer.<br><br>The intraoperative LightPath® images will be used to inform the operating surgeon about potentially detectable cancer at the margins of the excised specimen in an attempt to achieve better guided cancer surgery and complete tumour excision with clear resection margins. If a positive excision margin is detected on intraoperative LightPath® images, the operating surgeon will take a cavity shaving of the corresponding margin, provided more tissue can be taken.<br>Subsequent LightPath® imaging of the cavity shaving will inform the operating surgeon on the margin status of the shaving. If a positive excision margin is detected on intraoperative LightPath® images, the operating surgeon will take a further cavity shave of the corresponding margin, provided more tissue can be taken.<br><br>The resection margin status of the WLE specimen and cavity shavings (if any), as assessed by LightPath® CLI + FAR imaging will be compared with final histopathology results. A positive margin on histology will be defined as<br>• Invasive carcinoma: positive: <1mm; negative ≥1mm<br>• Ductal carcinoma in situ (DCIS) (if present): positive: <2mm; negative ≥2mm.

**Radio-pharmaceutical administration**	Subjects will receive an intravenous injection of 250 MBq (±10%) of ^{18}F-FDG prior to surgery. LightPath® images should be taken approximately 145 minutes post-administration.
**Length of Study**	Subjects included in the clinical study will be evaluated at screening and enrolled into the study. Data will be collected until the patient's surgical treatment is complete.
**Primary Outcome Measure**	Agreement between margin status of WLE specimens as determined by intraoperative CLI + FAR LightPath® imaging and post-operative histopathology.
**Secondary Outcome Measures**	• Agreement between margin status of cavity shavings as determined by intraoperative CLI + FAR LightPath® imaging and post-operative histopathology.<br>• Agreement between margin status of WLE specimens as determined by intraoperative CLI + FAR LightPath® imaging and routine intraoperative specimen x-ray.<br>• Re-operation rate within the study cohort compared to general breast cancer population undergoing a WLE.<br>• Time added to operating procedure.
**Sample Size**	Sixty (60) female participants.
**Power Calculation**	Using an estimate of the incidence of positive excision margins on histopathology as 20%, with 95%-confidence (alpha = 0.05 two-sided) and with 10% precision, a sample size of 54 patients will provide sufficient power to detect sensitivity of 95% and specificity of 90% (Cameron and Baldock 1998). To allow for any potential drop-out or non-analysable subjects a total sample size of 60 patients will be enrolled to attain N = 54 patients in the analysis.
**Recruitment period**	24-month recruitment period
**Inclusion Criteria**	• Female subjects ≥18 years of age with a diagnosis of invasive breast cancer scheduled to undergo BCS<br>• Subjects who are able to give voluntary, written informed consent to participate in this study<br>• Subjects who are able to understand this study and are willing to complete all the study assessments

	• Female subjects of childbearing age must have a negative pregnancy test (by Beta human chorionic gonadotrophin (β-HCG) qualitative analysis) or must have had a history of a surgical sterilisation or must give history of no menses in the past twelve months.
**Exclusion Criteria**	• Subjects who have had surgery to the ipsilateral breast in the past 12 months<br>• Subjects who have had radiotherapy to the ipsilateral breast<br>• Subjects who have a known hypersensitivity to ^{18}F-FDG<br>• Subjects who are pregnant or lactating<br>• Subjects who have an existing medical condition that would compromise their participation in the study
**Study Procedures**	Female participants with a diagnosis of invasive breast cancer scheduled to undergo breast-conserving surgery with or without sentinel lymph node biopsy (SLNB) or axillary lymph node dissection (ALND) will be screened and entered into the study if eligible.<br>All participants will receive an intravenous injection 250 MBq (±10%) of ^{18}F-FDG prior to surgery. LightPath® images will be acquired within approximately 145 minutes following administration.<br><br>BCS will be performed as per standard of care, with the addition of intraoperative LightPath® CLI + FAR imaging.<br><br>Following resection, the WLE specimen and cavity shavings (if any), will be assessed using the LightPath® Imaging System. The intraoperative LightPath® CLI + FAR images will be used to inform the operating surgeon about potentially detectable cancer at or close to the excision margins of the excised specimen in an attempt to achieve better guided cancer surgery and complete tumour excision with clear resection margins. If a positive excision margin is detected on intraoperative LightPath® images, the operating surgeon will take a cavity shaving of the corresponding margin, provided more tissue can be taken.<br>Subsequent LightPath® imaging of the cavity shaving will inform the operating surgeon on the margin status of the shaving. If a positive excision margin is detected on intraoperative LightPath® CLI + FAR images, the operating

	surgeon will take a cavity shave of the corresponding margin, provided more tissue can be taken.<br><br>All LightPath® images will be acquired within approximately 145 minutes post-injection.<br>The WLE specimen and cavity shavings (where performed) will undergo standard of care histopathological analysis.<br>Participants will return for a study completion visit, coinciding with their standard of care surgical follow-up for the MDT decision whether to re-operate (1-2 weeks after completion of the initially planned surgery).
**Data Analysis for Outcome Measures**	All patients who pass screening will be included in the intention to treat (ITT) population.<br><br>The per protocol (PP) population is defined as all patients who completed BCS and study procedures as per protocol description.<br><br>The primary endpoint will be analysed based on the PP population.<br><br>The secondary endpoints will be analysed using the PP population and the ITT population.<br><br>The primary analysis of the study, including analysis of all available study outcomes (primary, secondary and exploratory where data is available), will be conducted after all patients have completed the follow-up visit.<br><br>Subgroup analysis will be conducted to assess the impact of important subgroups (e.g., breast cancer subtypes, invasive/*in situ*) on the performance of intraoperative LightPath® CLI + FAR imaging.<br><br>All outcome measures will be summarised descriptively.
**End of Study Date**	Last patient last visit is anticipated no later than August 2025 and we will require an additional 12 months for analysis of the data before end of study can be declared.

2.4. Schedule of Events

Procedures	Screening	Day of Surgery	Discharge	Study Completion at MDT decision on re-operation (1-2 weeks)
Visit	*1*	*2*	*2*	*3*
Informed Consent	X			
Inclusion/Exclusion	X			
Demographics, Medical History	X			
Biomarkers (ER/PR/HER2/other, according to local practice)	X			
Injection of ^{99m}Tc		X		
Injection of ^{18}F-FDG		X		
Standard-of-care surgical procedure		X		
Intra-operative specimen x-ray analysis of WLE specimen as per standard-of-care		X		
LightPath® imaging of WLE specimen		X		
Cavity shaving if indicated by LightPath® Imaging and/or specimen x-ray		X		
LightPath® imaging of cavity shaving, if applicable		X		
Histopathology analysis of WLE specimen and cavity shavings as per standard-of-care		X		
Study related Adverse Events		X	X	X
Multidisciplinary team decision on re-operation (*within 1-2 weeks*)				X

3. Background Information

3.1. Wide Local Excision for Breast Cancer

Cancer is the second largest cause of death and morbidity in Europe, with more than 3.9 million new cases diagnosed each year. The most commonly occurring cancers are those of the female breast, with over 523,000 new cases diagnosed in 2018 (Ferlay et al 2018). The most effective treatment strategy for most forms of cancer is early detection followed by surgery and adjuvant therapy. Breast cancer screening has resulted in smaller lesions being identified at an earlier stage of the disease. This, together with patient preference, has resulted in an increase in the number of breast-conserving surgical procedures such as wide local excision (WLE).

The success of WLE depends on the complete excision of the tumour during the initial surgical procedure. Positive surgical margins represent a high risk for adverse clinical outcome in WLE. Previous studies have reported positive resection margins in 20% to 40% of the participants who underwent WLE (Pleijhuis et al 2009). Re-operation rates due to positive surgical margins following WLE reported in the literature range from 17-68%, with one published retrospective study reporting 20% of women having at least one re-operation within 3 months of WLE (Jeevan et al 2012). In addition to repeat operations, the consequences of positive surgical margins include delayed adjuvant treatment, poorer cosmesis and functional outcomes, emotional distress, and financial cost.

Tumours fail to be completely excised because the surgeon only has the visual appearance of the tumour, palpation and specimen x-ray to differentiate malignant from benign tissue. A standardised approach to margin assessment can reduce residual disease and likelihood of re-excision (Bolger et al 2015, Merrill et al 2016). Currently available technologies for image-guided cancer surgery either have low sensitivity (for example, ultrasound, optical coherence tomography) or require lengthy regulatory development of novel contrast agents (e.g., targeted fluorescence or photoacoustic imaging). A number of techniques have been investigated including frozen section analysis and touch imprint cytology. However, no single technique has been accepted as standard of care due to issues of practicality, increased surgery time, limited clinical utility, limited sensitivity and specificity and cost (National Institute for Health and Care Excellence (NICE). (2009)). Consequently, less than 5% of hospitals that perform WLE currently utilise intra-operative cytology or frozen section pathology analysis of tumour margins (Bydlon et al 2012).

Optical imaging has been considered for intra-operative imaging since the original use of fluorescein in 1947 (Moore 1947). It offers an attractive solution to the problem of intra-operative tumour margin assessment, as it is fast and non-destructive. A variety of different methods of optical imaging have been proposed, including intra-operative radiography, radiofrequency spectroscopy, Raman spectroscopy, fluorescence spectroscopy and optical coherence tomography. Each has limitations, either in sensitivity and specificity, shallow penetration, poor definition or time and cost to develop targeted fluorescent agents.

In conclusion, a key problem that currently affects the success of WLE is the lack of accurate, real-time techniques to assess tumour margins intra-operatively. Therefore, there is a clear unmet need for new intra-operative techniques that can accurately and adequately assess tumour margins, thereby reducing the number of patients that need to undergo a re-operation.

3.2. LightPath® Imaging

The LightPath® Imaging System is an *in vitro* diagnostic device intended to detect the location and distribution of positron-emitting radionuclides within excised surgical specimens (Grootendorst et al 2017). As the uptake of the PET imaging agent is greater in malignant cells than in healthy tissue, the tumour emits beta particles at a higher rate and intensity and can therefore be differentiated from surrounding healthy breast tissue. The LightPath Imaging System can detect the beta particle directly through Cerenkov luminescence imaging (CLI) or by measuring scintillations through Digital Autoradiography (DAR).

3.2.1. Cerenkov Luminescence Imaging (CLI)

CLI is an imaging modality that can provide real-time molecular imaging during surgery (Liu *et al*., 2012). CLI is based on the phenomenon that beta-emitting radiopharmaceuticals also generate light in the visible range, albeit at ultra-weak levels (Robertson et al, 2009). CLI enables optical imaging of commonly used PET radiopharmaceuticals. CLI combines the advantages of optical imaging (i.e., low cost and small form factor) with those of PET imaging (i.e., high diagnostic sensitivity and specificity).

3.2.2. Flexible Autoradiography (FAR)

With FAR the beta particles are detected indirectly. This is achieved by detecting light pulses (scintillations) produced when beta particles emitted by PET imaging agents are absorbed by a scintillating film placed over the surgical specimen (Vyas *et al.* 2018). The potential added value of FAR is an increased signal intensity and reduced detection of artefacts (Olde Heuvel et al., 2018). The feasibility of intraoperative FAR LightPath® imaging in breast cancer has been shown in a previous study by Jurrius *et al.,* 2021.

3.2.3. Benefits

Potential benefits intraoperative CLI + FAR LightPath® Imaging to the patients include:

- Reduction of re-operation rates
- Rapid molecular imaging in the operating room
- No complex sample preparation required
- Compatible with commercially available PET imaging agents

4. Administration of Radiopharmaceuticals

4.1. Administration of ^{18}F-fluorodeoxyglucose (^{18}F-FDG)

4.1.1. Rationale

The LightPath® Imaging System is an *in vitro* diagnostic device intended to detect the location and distribution of positron-emitting radionuclides within excised surgical specimens. For this clinical study, this is achieved by detecting Cerenkov light from and scintillations produced by a scintillating film when struck by beta particles emitted by the nuclear radiotracer ^{18}F-FDG in subjects with breast cancer.

4.1.2. Dose

Subjects will receive an intravenous injection of 250 MBq (±10%) of ^{18}F-FDG prior to surgery. The administered activity will be determined on a per subject basis.
^{18}F-FDG has a radioactive half-life of 110 minutes.

All LightPath® Images should be acquired approximately 145 minutes post injection of ^{18}F-FDG.

4.1.3. Regulatory Status

^{18}F-FDG is licensed by the appropriate regulatory authorities in UK (Medicine and Healthcare Product Regulatory Agency) as an approved medicinal product for this use.

4.1.4. Additional Information

Administration of ^{18}F-FDG does not interfere with subsequent histopathology analysis.

4.2. Administration of Technetium 99m (^{99m}Tc tracer)

4.2.1. Rationale

For subjects who are undergoing WLE plus SLNB, ^{99m}Tc tracer is administered in the peri-areolar region with an activity ≤40 MBq, as per local standard of care. In previously conducted studies on CLI and FAR, all SLNs were successfully identified using 37 - 150 MBq of ^{99m}Tc tracer in subjects who received ≤5 MBq/kg of ^{18}F-FDG (Grootendorst et al 2017, Jurrius et al., 2021).

4.2.2. Dose

Subjects will receive up to 40 MBq ^{99m}Tc tracer prior to the administration ^{18}F-FDG, as per standard-of-care. ^{99m}Tc tracer has a radioactive half-life 6.02 hours.

4.2.3. Regulatory Status

^{99m}Tc tracer is licensed by the appropriate regulatory authorities in UK (Medicine and Healthcare Product Regulatory Agency) as an approved medicinal product for this use.

4.2.4. Licence Requirements

The Nuclear Medicine department will ensure all the appropriate local licence requirements in relation to radiation requirements are in place prior to the commencement of the clinical study.

4.2.5. Radiation Safety

Prior to starting the study, all staff will have received training sessions in accordance with the hospital Nuclear Medicine and Radiation Safety policies and procedures. This is to ensure all staff are familiar with the radiation control procedures, occupational risks, and to learn how to minimise exposure without compromising patient care.

4.2.6. Handling of Radioactive Materials

All staff will follow local Standard Operating Procedures (SOPs) and ‘systems of work’ to ensure that all exposures to radioactivity are "as low as reasonably practicable" (ALARP), in accordance with IRR99 Regulation 8 and applicable local regulations.

A risk assessment will be performed prior to the study starting and appropriate procedures put in place. Thorough contamination monitoring will be completed in areas in which radionuclides have been present.

5. Imaging device

5.1. Imaging system

LightPath® Imaging System procured from LightPoint Medical Ltd.

5.2. Regulatory Status of Product

The LightPath® Imaging System is a CE marked *in vitro* diagnostic device.
^{18}F-FDG is a routinely used Positron Emission Tomography (PET) / Computed Tomography (CT) radiopharmaceutical

5.3. Description of Device

LightPath® Imaging System is an *in vitro* diagnostic device intended to detect the location and distribution of positron-emitting radionuclides within excised surgical specimens (see the LightPath®’s *Instructions for Use (IFU)*). This is achieved by detecting light, i.e., Cerenkov light emitted by the beta particle directly for CLI or scintillations produced by a scintillating film when struck by beta particles for Digital Autoradiography (DAR). As the uptake of the PET imaging agent is greater in malignant cells than in healthy tissue, the tumour emits more beta particles and can therefore be differentiated from surrounding healthy tissue. As beta particles can travel only approximately 3mm in tissue, the scintillating film will produce light only

when the tumour is approximately 3mm or less from the surface of the excised surgical specimen.

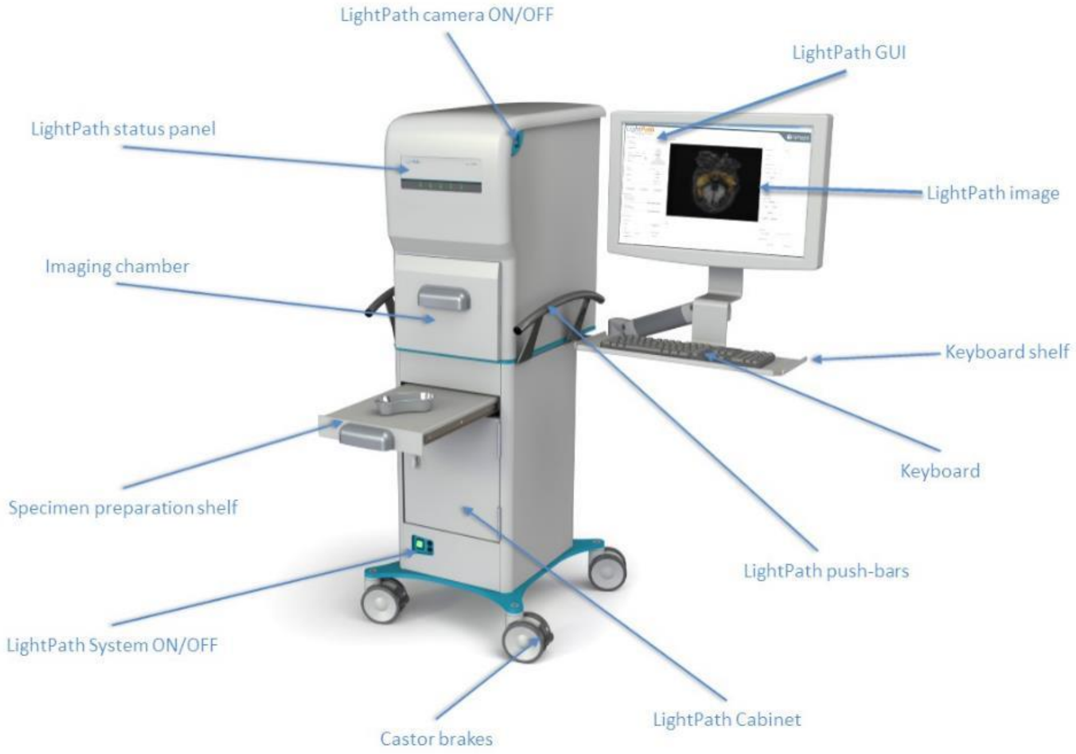

Figure 1. LightPath® Imaging System

The LightPath® Imaging System comprises of a light-tight specimen chamber incorporating an ultrasensitive electron multiplying Charge Coupled Device (emCCD) camera (Andor iXon Ultra 897, Andor Technology Plc), a camera for white light (WL) imaging, a sample stage and a source of WL. For FAR, the specimen is covered with a scintillating film; for CLI, no scintillating film is used. The specimen is positioned inside the light-tight box and the box is then illuminated using the WL source. The WL source is then extinguished so that the emCCD camera can begin to detect the LightPath® Image (see Figure 1).

Further information on the LightPath® Imaging System is provided in the IFU.

5.4. Packaging and Labelling of Device

The packaging and labelling for each device are detailed in the Investigator's Brochure (IB).

5.5. Device Training

The LightPath® Imaging System has been purchased by GSTT from LightPoint Medical Ltd., it has been installed on site and members of the research team have been trained.

6. Study Objectives

The objective of this study is to evaluate the diagnostic accuracy of intraoperative Cerenkov luminescence imaging (CLI) plus flexible autoradiography (FAR) using the LightPath® Imaging System for intraoperative tumour margin assessment compared to post-operative standard-of-care histopathology in women undergoing breast-conserving surgery for breast cancer. This will be assessed by examining the following outcome measures:

6.1. Primary outcome measure

- Agreement between margin status of WLE specimens as determined by intraoperative CLI + FAR LightPath® imaging and post-operative histopathology.

6.2. Secondary outcome measures

- Agreement between margin status of cavity shavings as determined by intraoperative CLI + FAR LightPath® imaging and post-operative histopathology.
- Agreement between margin status of WLE specimens as determined by intraoperative CLI + FAR LightPath® imaging and routine intraoperative specimen x-ray.
- Re-operation rate within the study cohort compared to general breast cancer population undergoing a WLE at Guy's Hospital.
- Time added to operating procedure.

7. Study Design

7.1. Overview of Study Design

This study is a prospective, single arm interventional study.

7.2. Risks and Benefits

The LightPath® Imaging System has undergone comprehensive pre-clinical testing, which demonstrates proof of concept and safety for its intended use. A full Risk Management Review has been conducted in accordance with ISO 14971:2007. Clinical studies have been conducted to evaluate the LightPath® Imaging System, amongst which is a multicentre study in women undergoing breast-conserving surgery (Jurrius et al., 2021).

7.2.1. Risks associated with LightPath® directed surgical procedures

As with all surgical procedures this carries a risk, which has been minimised by limiting the cavity shavings to a maximum thickness of 10mm. In perspective, the risk added by extra cavity shaving is expected to be small compared to the overall risk of undergoing further surgery for breast cancer.

7.2.2. Risk to participants

The LightPath® Imaging System is non-radiation emitting. Risks to participants in this study are associated with radiation exposure from the ^{18}F-FDG and its administration procedure. In addition, the LightPath® images may lead to extra cavity shavings.

7.2.3. Radiation exposure associated risks from ^{18}F-FDG

The effective dose to the subject from 300 MBq ^{18}F-FDG (an even higher activity than administered in the study) is approximately 6 mSv. This is comparable to 2.2 years' annual background radiation dose (2.7 mSv/year) from natural sources, or the radiation dose from a typical chest CT scan (6.6 mSv). Based on data published by the UK Health Protection Agency (Wall et al. 2011), the total lifetime cancer risk in females from an effective dose of 6.4 mSv (total study radiation dose) ranges from 0.054% at age 20 to 0.011% at age 70.

The International Commission on Radiological Protection (ICRP 62) categorises 1-10 mSv in Risk Category Class IIb. According to ICRP, this Risk Category requires Intermediate to Moderate Social Benefit, in particular, "benefits directly aimed at the cure or prevention of disease" (
Verbruggen et al., 2008). The clinical study objectives support this aim and hence, the radiation exposure is within the ICRP limit.

The total lifetime cancer risk for females aged 18 years and older is still small and within the ICRP limit (see Table 1). The inclusion criteria in this study state that females of childbearing age must have a negative pregnancy test (by Beta HCG qualitative analysis) or must have had a history of a surgical sterilisation, or must give history of no menses in past twelve months. Pregnant or lactating subjects are excluded from the clinical study. The added risk of heritable effects for female subjects of reproductive potential is very low; 1 in 10,000 to 1 in 100,000 (Wall et al. 2011, HPA –CRCE-028).

Table 1. Added risk of cancer from the Total Research Protocol Dose (TRPD) of 6.4 mSv.

Age	% risk	Risk
10-19	0.070	1 in 1400
20-29	0.054	1 in 1800
30-39	0.044	1 in 2300
40-49	0.037	1 in 2700
50-59	0.028	1 in 3600
60-69	0.020	1 in 5000
70-79	0.012	1 in 8700
80-89	0.004	1 in 22,300

7.2.4. Radiation exposure associated risks from ^{99m}Tc-nanocolloid

SLNB is currently routinely performed on breast cancer patients undergoing WLE who are clinically and radiologically lymph node negative. The procedure may vary between institutions, but is typically performed using 'the combined technique'. An

injection of 20 - 40 MBq of Technetium 99m (^{99m}Tc) tracer is given pre-operatively, and/or blue dye is injected intraoperatively. The SLNs are localised using a handheld gamma probe (such as Europrobe 3, Euromedical Instruments, Le Chesnay, France) and/or visually (i.e., blue discolouration).

In this study, the presence of ^{18}F-FDG will result in a greater than normal background signal being detected by the handheld gamma probe, potentially masking low activity SLNs.

To mitigate the risk of ^{18}F-FDG interfering with gamma probe detection of SLNs, the administered activity of ^{99m}Tc tracer for SLNB can be increased to up to 40 MBq as a one-day protocol for SLNB. Furthermore, a collimator will be attached to the gamma-probe, to block perpendicular gamma photons and allow for directional detection of SLNs. With this administered activity and the addition of a collimator, the ^{99m}Tc signal in the SLN has been shown to be sufficient to allow clear differentiation of the SLN from the ^{18}F-FDG background signal. The increased dose of 150 MBq allowed successful SLNB in all subjects in the clinical study of LightPath® in breast cancer conducted at Guy's Hospital in London, UK (LPM-001, ClinicalTrials.gov Identifier NCT02037269). A multicentre trial in Poland showed that a successful SLNB procedure may be achieved with an injected ^{99m}Tc activity as low as 37 MBq (LPM-007, ClinicalTrials.gov Identifier: NCT02666079) (Jurrius et al., 2021).

7.2.5. Combined radiation exposure associated risk of ^{18}F-FDG and ^{99m}Tc

The maximum overall effective dose from ^{18}F-FDG and ^{99m}Tc-nanocolloid combined is 6.4 mSv and this remains within ICRP Risk Category Class IIb.

7.2.6. Risk to staff

Radiation safety of theatre staff will be overseen by the GSTT Radiation Safety Team. Data on radiation dose using ^{18}F-fluorodeoxyglucose (^{18}F-FDG) at a higher dose in an earlier study (LPM-001, ClinicalTrials.gov Identifier NCT02037269), and data from a multicentre study on similar doses (LPM-007, ClinicalTrials.gov Identifier: NCT02666079) are available (Grootendorst et al., 2017; Jurrius et al., 2021) and indicate that staff radiation exposure is low. In the previous study, surgeons received the highest dose: up to 74 (mean 34) μSv per procedure (Table 2). This was less than the dose that was initially expected and similar to 10 days of background radiation from living in London. All other staff groups including theatre nurses and the anaesthetist had much lower exposure; all below 18 μSv, or 2 days' background radiation.

Table 2. Measured effective radiation doses by occupation from 22 surgical procedures during ^{18}F-FDG guided surgery (Grootendorst et al 2017).

Staff group	*n*	Mean effective dose per procedure ± SD (µSv)	Range (µSv)	Estimated no. of procedures per individual per year* (ICRP [20-mSv annual limit])	Estimated no. of procedures per individual per year* (USNRC [50-mSv annual limit])
Surgeon	46	34 ± 15	8–74	270	676
Anaesthetist	22	11 ± 5	0–18	1,111	2,778
Nuclear medicine technologist	22	9 ± 4	1–15	1,333	3,333
Anaesthetist assistant	22	6 ± 3	0–11	1,818	4,545
Trial coordinator	21	5 ± 2	1–10	2,000	5,000
Recovery nurse	43	4 ± 3	0–14	1,429	3,571
Scrub nurse	22	2 ± 1	0–5	4,000	10,000
Periphery nurse	23	1 ± 1	0–4	5,000	12,500
Research fellow	36	1 ± 2	0–13	1,538	3,846
Ward nurse	15	0	0–1	20,000	50,000
Tissue biobank practitioner	14	0	0–1	20,000	50,000

ICRP = International Commission on Radiological Protection; USNRC = U.S. Nuclear Regulatory Commission.
* Based on maximum effective dose per procedure per staff group.

A number of groups have studied the radiation exposure to the surgical team and theatre staff during radiopharmaceutical guided surgery (Table 3). Piert et al (2007) reported radiation dose rates ranging from 2.5 to 8.6 µSv/h for the surgeon to 0.8 µSv/h for the plastic surgeon in surgery following injection of 36–110 (mean 44) MBq of ^{18}F-FDG. The length of the procedures ranged between 2 and 6.25 hours. Povoski et al (2008) reported mean equivalent doses per case for the surgeon, anaesthetist, scrub nurse, post-operative nurse, circulating nurse, and pre-operative nurse as 164, 119, 92, 63, 54, and 48 µSv, respectively, with a mean injected activity of 699 MBq of ^{18}F-FDG. Moran et al 2014 and Nalley et al 2010 reported exposure levels of 37 and 8 µSv respectively for the surgeon and operating room staff with a mean ^{18}F-FDG activity of 287.5 MBq. Andersen et al (2008) reported mean equivalent doses in the range of 7.5 - 13.2 µSv for the surgeon and 2.5 µSv for other operating room staff, with a mean ^{18}F-FDG injected activity of 45 MBq. Heckathorne et al (2008) reported that for surgical procedures performed 1-3 hours after injection of 370 MBq of ^{18}F-FDG, the effective dose to the surgeon was 59 µSv and to the nursing staff 22 µSv.

All authors report the levels of exposure as being relatively low per case and consider that they allow for theatre personnel to participate in multiple cases and remain well below regulatory standards set for occupational radiation exposure limits. The levels of per procedure radiation exposure reported in the literature would be classified by ICRP 62 as Trivial (Class I), which specifies that "the level of benefit needed as the

basis for approval of investigations with risks or doses in category I will be minor and would include those investigations expected only to increase knowledge" (Verbruggen et al 2008). All authors advise on routine monitoring of radiation exposure of staff involved in ^{18}F-FDG guided surgical procedures.

The mean administered activity in the study by Povoski et al (2008) gave a mean measured dose rate (61 µSv/h) which suggests that a surgeon could perform approximately 100 study procedures per year and remain below the 6 mSv threshold requiring designation as a classified radiation worker under Regulation 20 of the Ionising Radiations Regulations 1999.

Table 3 Summary of published radiation exposure figures to staff during ^{18}F-FDG guided surgery

Publication	Injected activity of ^{18}F-FDG (MBq)	Time from injection to surgery (mins)	Time in operating room (Exposure time) (mins)	Mean dose equivalent per hour (µSv/h)		
				Surgeon	Nurse / theatre staff	Anaesthetist
Nalley et al.	287.5	228	Not reported	37.5	9.3	2.0
Povoski et al.	699	179	235	61	17	49
Heckathorne et al.	353	79	(48.5 - surgeon) (85.5 - nurse)	23	10.5	NR
Piert et al.	44	222.5	247.5	5.55	0.8	NR
Andersen et al.	45	65	15 – 450	13.2	2.7	NR

7.2.7. Benefits to Participants

This study offers the potential benefits of more complete cancer detection and surgery to participants. The data collected in this study may also potentially provide benefits for future patients by informing the further development of the LightPath® Imaging System for the intraoperative assessment of tumour margins and lymph node involvement.

7.3. Study Population

Women ≥18 years of age with invasive breast cancer scheduled for primary breast-conserving surgery with or without sentinel lymph node biopsy (SLNB) or axillary lymph node dissection (ALND).

7.4. Inclusion Criteria

- Female subjects ≥18 years of age with a diagnosis of invasive breast cancer scheduled to undergo BCS
- Subjects who are able to give voluntary, written informed consent to participate in this study
- Subjects who are able to understand this study and are willing to complete all the study assessments

- Female subjects of childbearing age must have a negative pregnancy test (by Beta human chorionic gonadotrophin (β-HCG) qualitative analysis) or must have had a history of a surgical sterilisation or must give history of no menses in the past twelve months.

7.5. Exclusion Criteria

- Subjects who have had surgery to the ipsilateral breast in the past 12 months
- Subjects who have had radiotherapy to the ipsilateral breast
- Subjects who have a known hypersensitivity to ^{18}F-FDG
- Subjects who are pregnant or lactating
- Subjects who have an existing medical condition that would compromise their participation in the study.

8. Study Procedures

8.1. Study Start and Training

The study will not commence until all the necessary approvals are in place and a formal initiation visit has taken place.

At the initiation visit, the PI will ensure that all study personnel are fully aware of all the study requirements.

8.2. Recruitment

Female patients ≥18 years of age with a diagnosis of invasive breast cancer, and scheduled to have breast conserving surgery (BCS) with or without SLNB or ALND, will be screened for enrolment in the study at the Breast Multidisciplinary Team Meetings (MDM) according to the criteria listed in sections 7.4 and 7.5 of this protocol. A member of the research team will then approach the patient, at the time of their clinical hospital visit, should the situation and the patient's emotional status allow it. Patients who display an interest in participating will be provided with a copy of the Patient Information Sheet (PIS). Patients who have been provided with the PIS will be approached by a member of the research team at their next visit or receive a telephone call to answer any questions that may arise. Patients are then invited to participate in the study, and informed consent will be obtained as outlined in section 8.3.

8.3. Informed Consent

Potential participants will be provided with written information concerning the study, advising them of the study requirements, and possible risks. Appropriately qualified healthcare staff trained in this protocol and GCP will ensure that potential participants understand the information provided, and review requirements and potential risks. Potential participants will have at least 48 hours in which to review this information.

Each subject will be informed that participation in the study is voluntary and non-participation will not affect their care. Subjects are free to withdraw from the study at any time irrespective of their initial consent.

Each subject will also give their permission for representatives of the Sponsor, auditor and regulatory authorities to review their hospital records for the purposes of source data verification.

Individuals agreeing to participate in the study will sign an informed consent form approved according to local regulations. All signed consent forms will be maintained in the investigator site file at the study site and a copy provided to the subject. Upon verification of eligibility and execution of informed consent, subjects will be assigned a unique identification code number, which will be used to identify the subject and maintain anonymity of personal data.

8.4. Baseline Data

Relevant demographic data, e.g., age, height and weight will be collected, the medical history, including the diagnosis for which surgery is indicated, tumour characteristics, e.g., size, grade, receptor status, focality, and any lymph node involvement will be recorded.

If a participant fails to fulfil any element of the inclusion and exclusion criteria, this will be documented and the signed consent form and completed inclusion/exclusion criteria retained by the Investigator. The participant will not be advanced any further in the study.

8.5. Concomitant Medication/Treatments

No concomitant medication and/or treatments will be withheld for any subject participating in this study. All concomitant medication and/or treatments will be recorded.

8.6. Day of surgery

8.6.1. Radiopharmaceutical administration

Prior to her surgery, the participant will be prepared for surgery in accordance with the local hospital standard of care. The following information/procedures will be completed on the day of surgery or the day before surgery by a designated team trained in administering radiopharmaceuticals:

- Administration of ^{99m}Tc tracer prior to the administration ^{18}F-FDG
- Administration of ^{18}F-FDG.

Administration of ^{18}F-FDG will be scheduled accordingly, to ensure the LightPath® images will be taken approximately 145 minutes post-injection of ^{18}F-FDG.

Work-up and preparation for surgery will be performed as per standard of care.

8.6.2. BCS only

Participants scheduled to have BCS will be administered ^{18}F-FDG intravenously in the Nuclear Medicine Department, approximately 145 minutes prior to CLI + FAR LightPath® imaging. BCS will be performed as per standard of care. Following resection, the WLE specimen and shavings (if any) will be examined intraoperatively using CLI + FAR LightPath® imaging. If the surgeons detect a positive signal, they will perform cavity shavings of the resection cavity area corresponding to the positive signal area. It will be at the surgeon's discretion to choose whether to act based upon the intraoperative LightPath® images.

8.6.3. BCS plus axillary surgery (SLNB or ALND)

Participants scheduled to have BCS plus SLNB will receive ^{99m}Tc and ^{18}F-FDG. ^{99m}Tc will be injected in the peri-areolar region and ^{18}F-FDG administered intravenously in the Nuclear Medicine Department, approximately 145 minutes prior to CLI + FAR LightPath® imaging. Blue dye might be injected intraoperatively according to the local protocol; BCS and axillary surgery will be performed as per standard of care. In patients undergoing an SLNB, a collimator attached to the gamma-probe will allow directional detection of lymph nodes and shield the probe from ^{18}F-FDG activity from the main tumour. Imaging procedures of the WLE specimen will be as described in section 8.6.2.

Sutures and surgical clips will be placed on the WLE specimen to record the anatomical orientation, as per standard practice. Based on the information on the LightPath® images additional sutures may be used to allow precise mapping between the histopathological analysis and CLI + FAR LightPath® imaging. Information on the surgical procedure will be recorded on the study case report form (CRF).

8.6.4. LightPath® assessment

The WLE specimen will be examined by CLI + FAR LightPath® imaging at the time of the surgical procedure. All LightPath® images will be performed within approximately 145 minutes post-injection of ^{18}F-FDG. If imaging is delayed for any reason, the reason must be recorded on the CRF. Imaging must be performed without unnecessary delay and following the acquisition times set out in the IFU, in order to avoid any degradation in specimen quality prior to transfer to the tissue bank / pathology department for routine analyses.
The operating surgeon will interpret the recorded CLI + FAR LightPath® Images. It will be at the surgeon's discretion to choose how to act on the CLI + FAR LightPath® images.

8.6.5. Imaging of the WLE specimen

The WLE specimen will be placed in the LightPath® specimen holder to maintain orientation during examination. Once the WLE specimen has been fixed in the

specimen holder it will be positioned inside the light-tight imaging chamber of the LightPath® System and imaged using the white-light video camera to assess correct positioning of the sample in imaging system. Once positioned correctly, CLI LightPath® Imaging will be performed using the LightPath®'s emCCD camera. Subsequently, the specimen will be covered with a scintillating film for FAR imaging with the LightPath®'s emCCD camera.

8.6.6. Imaging of the cavity shavings

CLI + FAR LightPath® imaging of cavity shavings (if any) is performed similar to imaging of the WLE specimen, as described above.

8.6.7. Radiation safety

Prior to starting the study, all staff will have received training sessions in accordance with the hospital Nuclear Medicine and Radiation Safety policies and procedures. This is to ensure all staff are familiar with the radiation control procedures, occupational risks, and to learn how to minimise exposure without compromising patient care.

8.6.8. Histopathological assessment

The WLE specimens and cavity shavings will then be examined according to the standard of care histopathology analysis as per the routine pathway at Synlab, Histopathology laboratories, St. Thomas' Hospital. Details of tumour dimensions along with all other histopathology minimum dataset items will be recorded. The distance from the tumour to the radial margins (medial, lateral, superior, inferior, posterior and anterior) will be recorded.

For the histopathology central assessment of the WLE, margins are defined (Toss et al 2017) as either:

- Invasive carcinoma – positive <1mm; negative ≥1mm
- DCIS - positive: <2mm; negative ≥2mm

8.6.9. Safety

Details of any adverse events will be recorded.

8.7. Follow-up

Adverse events will be recorded until discharge, or for 24 hours after the first study procedure, whichever is sooner.

Participants will return for a study completion visit. Participants will return following the MDT recommendation whether to re-operate (1-2 weeks after completion of the initially planned surgery).

8.8. Study Duration

8.8.1. Duration of Subject Participation

The point of enrolment in the study is the point at which the subject provides written informed consent and participation in the treatment phase ends at the conclusion of surgery. Adverse events will be recorded until discharge, or for 24 hours after the first study procedure, whichever is sooner. Participants will return for a study completion visit at the time of the MDT decision whether to re-operate (1-2 weeks after completion of the initially planned surgery).

8.8.2. Duration of the Clinical Study

It is anticipated that recruitment of up to 60 subjects will take approximately 24 months.

8.9. Withdrawal of Subjects

A subject may withdraw from the study at any time. If a subject chooses to withdraw their consent, no additional study related procedures will be performed. Subjects will continue to receive standard of care treatment and follow-up. The Investigator should endeavour to find out the reasons, if any, for withdrawal from the subject and document this in the CRF.

8.10. Handling of Radioactive Materials

All staff will follow local systems of work to ensure that all exposures are "as low as reasonably practicable" (ALARP), in accordance with the Approved Code of Practice and Guidance L121 - Ionising Radiations Regulations 2017. It is unlikely that the removed tissue samples will be a source of radiation risk to the staff handling them, but care may need to be taken when disposing of clinical waste generated from samples. A risk assessment will be performed prior to the study starting and appropriate procedures put in place. Thorough contamination monitoring will be completed in areas in which radionuclides have been present.

9. Study Monitoring and Auditing

The Chief Investigator will be responsible for the ongoing management of the study. The Sponsor will monitor and conduct audits on a selection of studies in its clinical research portfolio. Monitoring and auditing will be conducted in accordance with the UK Policy Framework for Health and Social Care 2017 and in accordance with the Sponsor's monitoring and audit procedures.

10. Adverse Events Definition and Reporting

10.1. Adverse Event Definition

An adverse event (AE) is any untoward medical occurrence in a participant or participant administered a pharmaceutical product (or surgery in this study) and which does not necessarily have a causal relationship with this treatment. An AE can therefore be any unfavourable and unintended sign (including an abnormal laboratory finding), symptom, or disease temporally associated with the use of a

medicinal product as part of a clinical study, whether or not related to the medicinal product (or surgery).

All AEs, either observed by the Investigator or reported by the participant, must be reported immediately, and no later than 24 hours of becoming aware of the event, to the Sponsor. To report an AE, the Principal Investigator (PI) will complete an AE report form or other relevant form located in the Case Report Form. The report will include the following information: nature, severity, date of onset, duration, course and history of the adverse event/adverse device effect and causality.

Recording of AEs will begin with the commencement of the first study procedure. As all study procedures are completed prior to the end of surgery and the radiopharmaceuticals have short physical half-lives, AE recording will conclude when the subject is discharged from hospital, or 24 hours following the first study procedure (whichever is sooner). Any adverse event identified in this period considered by the Principal Investigator (PI) to be related to a study procedure will be followed until resolution or for 30 days, whichever is sooner.

10.2. Serious Adverse Event Definition

A Serious Adverse Event (SAE) is defined as an adverse event that:

- led to death
- led to a serious deterioration in the health of the participant, that either resulted in:
  - a life-threatening illness or injury
  - a permanent impairment of a body structure or a body function
  - in-patient hospitalisation or prolongation of existing hospitalisation (defined as greater than 24 hours in hospital)
  - medical or surgical intervention to prevent life-threatening illness or injury or permanent impairment to a body structure or a body function
- led to foetal distress, foetal death or a congenital abnormality or birth defect.

Planned hospitalisation for a pre-existing condition, or a procedure required by the protocol, without serious deterioration in health, is not considered a SAE.

10.3. Anticipated Adverse Events

Within the context of BS EN 13612 (2002) and with the knowledge that the excision of tissue will not be outside of the normal practice as deemed appropriate by the PI, the LightPath® Imaging System cannot be associated with adverse events.

The radiopharmaceuticals used in this study are commonly administered at doses required by this protocol with no reported safety issues. Therefore, no AEs related to the administration of radiopharmaceuticals are anticipated.

The following are known complications of breast surgery, sentinel lymph node biopsy (SLNB) and axillary lymph node dissection (ALND) (Vitug and Newman 2007), and are anticipated procedure-related AEs:

- Wound infection (including cellulitis and abscess formation)
- Seroma
- Haematoma
- Pain
- Lymphoedema
- Neurosensory disturbance in the upper limb
- Allergic reaction to blue dye

The participants may have additional cavity shavings compared to standard of care surgery at the Investigator's discretion. Risks added by this minor addition to the already major procedures of standard of care surgery are expected to be minimal.

10.4. Adverse Event Severity

AE severity will be assessed according to the Common Terminology Criteria for Adverse Events (CTCAE) version 4.0 (U.S. Department Of Health And Human Services 2009):

- Grade 1 Mild; asymptomatic or mild symptoms; clinical or diagnostic observations only; intervention not indicated.
- Grade 2 Moderate; minimal, local or non-invasive intervention indicated; limiting age-appropriate instrumental activities of daily living.
- Grade 3 Severe or medically significant but not immediately life-threatening; hospitalisation or prolongation of hospitalisation indicated; disabling; limiting self-care activities of daily living.
- Grade 4 Life-threatening consequences; urgent intervention indicated.
- Grade 5 Death related to AE

It is important to distinguish between serious and severe AEs. Severity is a measure of intensity whereas seriousness is defined by the criteria under 10.2. An AE of severe intensity may not be considered serious.

10.5. Relationship to Study Procedures

The following definitions will be used to assess the relationship between an AE and the study procedures, radiopharmaceutical, or operational procedure. The CI will specify in each case if the event relates to the study procedures, to the radiopharmaceutical, and / or to the surgery procedure.

Not Related
The event can be determined with certainty to have no relationship to the study procedures.

Unlikely to be Related
There is no evidence for the causal relationship between the study procedures and the AE. The event was likely produced by other factors such as the subject's clinical state, surgical / therapeutic intervention, or concomitant therapy but for which relationship cannot be ruled out.

Possibly Related
The event has a reasonable temporal relationship to the study procedures and follows a known response pattern to the study procedures. However, a potential alternate aetiology may be responsible for the event, such as the subject's clinical state, surgical / therapeutic intervention, or concomitant therapy.

Probably Related
The event follows a reasonable temporal sequence from the time of the study procedures and/or follows a known response pattern to the study procedures and was unlikely to have been produced by other factors such as the subject's clinical state, surgical / therapeutic intervention, or concomitant therapy.

Related
The event follows a temporal sequence from the time of the study procedures and follows a known response pattern to study procedures and either occurs immediately following study procedures, or improves on stopping the study procedures, or reappears on repeat exposure to the study procedures.

10.6. Adverse Event Reporting

AEs occurring from the first study procedure until the end of follow-up will be reported by completing the relevant section of the CRF. AEs judged by the CI to be related to the standard care of the participant and unrelated to any study procedure do not require to be reported.

The following information will be recorded in the adverse event section of the CRF:

- The signs, symptoms, or diagnosis of the event
- The date and time of onset and resolution of the event
- The adverse event severity using the criteria outlined above
- The relationship of the event to the study procedures as outlined above
- The seriousness of the event as outlined above;
- A list of any required therapy, medication, treatment, or diagnostic procedure
- Any additional data which might be relevant to the event.

The CI is responsible for the appropriate medical management of all adverse events and for the personal safety and wellbeing of the participants.

In addition, a positive sterility test result post-release for a batch which has been administered to patients will be classified as an Adverse Event of Special Interest (AESI). For this reason, infections that could be signs and symptoms possibly related

to bacterial endotoxin will be considered AESI and be immediately reported to the Sponsor even if non-serious. All AESI must be reported in the same manner as SAE reporting and will be handled in the same way.

10.7. Serious Adverse Event Reporting

Any serious adverse event must be reported to the sponsor and, where required, the relevant ethics committee within 24 hours of the onset of the event. Full details of the event, treatment, and an assessment of the relationship to study procedures must be provided in the report.

All serious adverse events will be followed until they are resolved or for 30 days after the subject's participation in the clinical study ends.

The CI should institute appropriate therapeutic and follow-up measures in accordance with good medical practice but should notify the monitor of such actions and record them in the subject's Case Report Form.

10.8. Device Deficiencies

Device deficiencies are defined as 'Any inadequacy of the study device with respect to its identity, quality, durability, reliability, safety or performance'. Device deficiencies include malfunctions, user errors and inadequate labelling. All device deficiencies will be recorded in the CRF. Any deficiency of the device that is considered to constitute a hazard to the safety of users must be reported to the Sponsor within 24 hours of identification.

11. Data Collection, Processing and Statistical Analysis

11.1. Completion of Data Collection Forms

The Chief Investigator (CI) must maintain required records on all study subjects. Data for this study will be recorded in the subject's medical records, study-specific worksheets and on Case Report Forms (CRFs) provided by the sponsor in accordance with the parameters set forth in ICH Topic E6 for GCP (1.5.96) Guidelines - Responsibilities of Sponsor, Monitor and Investigator, and with 21 CFR Part 812. All data on the CRFs should be recorded from appropriate source documentation. No personal identifiable data will be entered on CRFs. Subjects will be identified only by their unique identification code number. All data recorded on all documents will be regarded as confidential.

The CI must record the subject's participation in this clinical study in the subject's medical notes. In addition, the CI must keep a separate list of all subjects entered into the clinical study showing each subject's name, date of birth and assigned subject number (for identification purposes). A Subject Identification Log will also be provided in the Investigator Site File to record the subject's initials and assigned subject number.

11.2. Data Correction

Corrections to data entered on original CRFs must be made in the following manner:

- The correct data should be entered clearly replacing the incorrect entry but maintaining an appropriate audit trail.
- Completed CRFs should be ready for review by the monitor prior to study visit. The Sponsor will review the CRFs, evaluate them for completeness and accuracy, and ensure all corrections are made to either the CRF or other designated clarification form.

11.3. Review and Return of Completed Documentation

Upon study completion, or at any other time specified by the sponsor, a monitor will verify the source documentation records and review the data, conducting source document verification. Copies of all CRF pages will remain at the study site with any related data clarification forms (DCF).

11.4. Data Protection

The study sponsor, GSTT and KCL, will manage the clinical data in clinical study data management systems that comply with all applicable EU and national standards including International Conference on Harmonization (ICH) Good Clinical Practice Section 5.5 (Trial Management, Data Handling, and Record Keeping) and the UK Data Protection Act (2018).

All data, including imaging data files, will be anonymised before being entered into the study database or being transmitted to the sponsor. All data will be kept for at

least 5 years and backed up in accordance with GCP, national and EU legislation. Data protection and security will be ensured by the local data controller nominated for this task.

Access to the data will be limited to only the primary research team, with access via a key which will be safely kept in a locked draw in the oncology research offices at King's College London, F03 Bermondsey Wing, Guy's Hospital, Great Maze Pond, London, SE1 9RT

11.5. Record Retention

The investigator will maintain records for this study, which includes subject's CRFs, medical records, laboratory reports, signed informed consent forms, safety reports, plus all other pertinent data. The investigator will retain study medication disposition records, copies of CRFs (or electronic files), and source documents for the maximum period required by the country in which the study will be conducted, or the period specified by the sponsor, whichever is longer.

European Commission Directive 2005/28/EC requires that investigators and sponsors shall retain the essential documents for at least 5 years after completion of a clinical trial.

If the investigator relocates, retires, or withdraws for any reason from the study, trial records may be transferred to an acceptable designee, such as another investigator within the institution. Prior notice of such transfer will be provided in writing to the sponsor. The investigator must obtain written permission from the sponsor prior to disposing of any records.

11.6. Data Analysis

11.6.1. Analysed Population

All participants enrolled into the study will be assigned to the following analysis sets:

- <u>The intention to treat (ITT) analysis set</u>. This will comprise all patients assigned to treatment.

- <u>The Per Protocol analysis set</u>. This set will comprise all participants who complete the study according to the protocol and who were compliant with assessment procedures (i.e., completed follow-up visits in accordance with local practice). It includes only those participants whose WLE specimens, and cavity shavings (if any) specimens were imaged within the time-window of approximately 145 minutes post-injection.

Further sensitivity analysis and summaries will be conducted based on sub-sets of the per protocol analysis set: e.g., subsets based on breast cancer diagnosis prior to surgery (invasive/DCIS), age etc.

A summary of the rate of decision to re-operate in the "Delayed LightPath® image set" (any participant for whom LightPath® Image of WLE and any cavity shaving specimens is outside the optimal time-window) will be presented as these participants will be excluded from the per protocol analysis set.

11.6.2. Sample Size Determination

The primary endpoint of the study is to report the sensitivity and specificity of CLI + FAR LightPath® imaging for tumour detection compared to positive tumour detection using standard-of-care histopathology methodology (positive margin of a WLE sample).

Using an estimate of the incidence of positive excision margins on histopathology as 20%, with 95%-confidence (alpha = 0.05 two-sided) and with 10% precision, a sample size of 54 patients will provide sufficient power to detect sensitivity of 95% and specificity of 90% (Cameron and Baldock 1998). To allow for any potential drop-out or non-analysable subjects a total sample size of 60 patients will be enrolled to attain N = 54 patients in the analysis.

11.7. Data Analysis for Outcome Measures

Most data analysis will be descriptive only due to the non-comparative nature of this study. Continuous variables will be summarised descriptively by number of subjects, mean, standard deviation, minimum, and maximum. Categorical data will be summarised descriptively by frequencies along with associated percentages.

The primary outcome measure will be analysed on the per protocol analysis set. All other outcomes will be analysed based on the ITT set and on the per protocol analysis set (unless stated otherwise). The diagnostic performance of the LightPath® Imaging will be evaluated based on the primary endpoint analysis. Any other p-values presented from analysis of secondary endpoints will be used to aid interpretation and will not be adjusted for potential correlation between endpoints or multiple-testing.

Baseline participant characteristics will be summarized descriptively; continuous variables will be summarised descriptively by number of participants, mean, standard deviation, minimum, and maximum; categorical data will be summarised descriptively by frequencies along with associated percentages.

11.7.1. Analysis of the Primary Outcome Measure

The primary outcome will be the diagnostic performance of intraoperative CLI + FAR LightPath® imaging, assessed using a confusion matrix to describe the relationship between the results of tumour margin assessment (positive or negative) with CLI + FAR LightPath® imaging and standard-of-care histopathology (positive or negative) (gold standard).

	Standard-of-care histopathology		
	Positive	Negative	Total

CLI + FAR LightPath® imaging	Positive	a (TP)	b (FN)	a+b
	Negative	c (FP)	d (TN)	c+d
	Total	a+c	b+d	n

Sensitivity = a/(a+c)
Specificity = d/(b+d)
Overall agreement = (a+d)/(a+b+c+d)
TP = true positive; FP = false positive; FN = false negative; TN = true negative

The primary outcome measures are sensitivity, specificity and overall accuracy.

The following diagnostic accuracy statistics will be presented (as a percentage together with 95% exact confidence intervals):

- Sensitivity - The proportion of subjects with positive margin confirmed by histopathology who have a positive result on LightPath® Imaging.
- Specificity - The proportion of subjects with clear margin confirmed by histopathology who have negative result on LightPath® Imaging.
- Overall accuracy - The proportion of subjects whose margin status is correctly classified by LightPath® Imaging
- Positive predictive value - The probability of positive margin in subjects with a positive result on LightPath® Imaging.
- Negative predictive value - The probability of clear margin in subjects with a negative result on LightPath® Imaging.
- Positive likelihood ratio (LR+) = sensitivity/1-specificity
- Negative likelihood ratio (LR-) = 1-sensitivity/specificity
- Diagnostic odds ratio (DOR) = (TP/FP)/(FN/TN)

As a sensitivity analysis, the primary analysis will be repeated using the ITT analysis set and for the combined per protocol set.

Analysis will be performed on the approximately 360 margins (60 patients x6 margins). The number of false positive, false negative, true positive, and true negative margins identified by intraoperative CLI + FAR LightPath® imaging will be reported.

11.7.2. Analysis of Secondary Outcome Measures

- Agreement between margin status of cavity shavings as determined by intraoperative CLI + FAR LightPath® imaging and post-operative histopathology will be assessed similarly to the primary outcome measure (section 11.7.1).
- Agreement between margin status of WLE specimens as determined by intraoperative CLI + FAR LightPath® imaging and routine intraoperative specimen x-ray will be assessed similarly to the primary outcome measure (section 11.7.1).

- Re-operation rate within the study cohort will be compared to the general breast cancer population undergoing a WLE at Guy's Hospital. The re-operation rate within Guy's Hospital is routinely recorded, audited and have remained consistent at 20 – 25% over many years.
  By recording the time required to intraoperatively image and assess each specimen, the average time added to the operating procedure can be determined.

11.7.3. Safety Analysis

Study-related Adverse Events
Adverse events will be coded according to coding dictionaries (the web based MedDRA version current at the time of coding) with relationship determined by the investigator. AEs will be classified by system organ class and preferred term. AE data will be listed individually, and each AE (based on preferred terminology) will be counted only once for a given participant. If the same AE occurs on multiple occasions, the highest severity and least complimentary relationship will be assumed. If two or more AEs are reported as a unit, the individual terms will be reported as separate experiences. Adverse events will be summarised based on the ITT analysis population and will be summarised descriptively by group by frequencies along with associated percentages.

11.8. Termination / Drop-out

Recruitment will continue until up to 60 participants have been imaged. It is anticipated that approximately 10% of participants may drop out of the study or not be evaluable for the primary endpoint. The sample size of n= 54 for the primary endpoint was selected including allowance of the assumption of 10% drop out. No other provision has been made for participant drop out. Patients who drop out will not be replaced.

12. Statement of Compliance

This clinical study will be conducted in accordance with the Declaration of Helsinki (2013), ICH Harmonized Tripartite Guideline for Good Clinical Practice R2 (2017), BS EN 13612:2002 and all applicable local and national regulations.

This clinical study may not commence at any study site until all required approvals / favourable opinions for that study site have been obtained from the appropriate institution, Ethics Committee and Competent Authorities (where applicable). Any additional requirement imposed by the Ethics Committee or Competent Authority shall be followed.

A copy of the written notification of study approval and associated documents, detailing statement of compliance, will be stored in the Clinical Trial Master File and the Investigator Site File. The CI agrees to promptly report to the institution

(hospital/clinic) and Ethics Committee all unanticipated problems involving risks to human participants or others.

12.1. Insurance

The study is co-sponsored by King's College London (KCL) and Guys and St Thomas' NHS Foundation Trust (GSTT). The sponsors will, at all times, maintain adequate insurance in relation to the study. KCL through its' own professional indemnity (Clinical Trials) & no fault compensation and the GSTT having a duty of care to patients via NHS indemnity cover, in respect of any claims arising as a result of negligence by its employees, brought by or on behalf of a study participant.

13. Quality Control and Quality Assurance

Investigators who participate in this clinical study and who sign the study documentation will seek to ensure that all participants included in the study are compliant with the requirements of the study at the point of entry and remain so during their participation in the study.

Repeated and serious failures in participant compliance will be addressed as they have the potential to affect both patient safety and study outcome and where necessary corrective and preventative actions will be taken.

14. Protocol Amendments

The CI agrees not to make any changes to, or deviate from, the protocol except when necessary to eliminate immediate hazards to human life.

Changes in the protocol may be made only by written amendment by the sponsor. Protocol amendments must be approved by the Ethics Committee and, if appropriate, the Competent Authority before implementation. Documentation of approval for such amendments must be stored in the Clinical Investigation Master File and the Investigator Site File.

14.1. Procedure for Reporting Deviations from the Protocol

All deviations from the protocol must be recorded and reported to the sponsor. A Protocol Deviation Form must be completed for each study protocol deviation. An Investigator must notify the sponsor and the Ethics Committee of any significant deviation from the protocol that was done to protect the life or physical wellbeing of a subject (medical emergencies). Such notice should be given within 24 hours of the emergency occurring.

15. Device Accountability and Storage

A LightPath® Imaging System has been procured from LightPoint Medical Ltd. and is stored in a safe and secure location within the KCL and GSTT premises, according to local guidelines. Regular electrical safety checks will be performed by the GSTT MEMS department, following the local regulations. LightPoint Medical Ltd. will perform system maintenance as and when required.

16. References

Andersen PA, Chakera AH, Klausen TL, Binderup T, Grossjohann HS, Friis E, Palnaes Hansen C, Schmidt G, Kjaer A, Hesse B (2008). "Radiation exposure to surgical staff during F-18-FDG-guided cancer surgery". Eur J Nucl Med Mol Imaging 35(3): 624-9.

Bolger JC, Solon JG, Khan SA, Hill AD, Power CP (2015). "A comparison of intra-operative margin management techniques in breast-conserving surgery: a standardised approach reduces the likelihood of residual disease without increasing operative time". Breast Cancer 22(3): 262-8.

Brix G, Nekolla EA, Borowski M, Noßke D (2014). "Radiation risk and protection of patients in clinical SPECT/CT". Eur J Nucl Med Mol Imaging 41 Suppl 1:S125-36.

Bydlon, TM, Barry, WT, Kennedy, SA, Brown, JQ, Gallagher, JE, Wilke, LG, Ramanujam, N (2012). "Advancing optical imaging for breast margin assessment: an analysis of excisional time, cautery, and patent blue dye on underlying sources of contrast". PloS ONE *7*(12), e51418.

Calvert M, Blazeby J, Altman DG, Revicki DA, Moher D, Brundage MD (2013). "Reporting of patient-reported outcomes in randomized trials: the CONSORT PRO extension". Jama. 2013;309(8):814-22.

Cescato R, Maina T, Nock B, Nikolopoulou A, Charalambidis D, Piccand V, et al (2008). "Bombesin receptor antagonists may be preferable to agonists for tumor targeting. Journal of nuclear medicine: official publication, Society of Nuclear Medicine 49: 318-26.

Cameron AR, Baldock FC. A new probability formula for surveys to substantiate freedom from disease. Prev Vet Med. 1998 Feb 6;34(1):1-17.

Dalm SU, Martens JW, Sieuwerts AM, van Deurzen CH, Koelewijn SJ, de Blois E, et al. (2015a). "In vitro and in vivo application of radiolabeled gastrin-releasing peptide receptor ligands in breast cancer. Journal of nuclear medicine : official publication, Society of Nuclear Medicine 56: 752-7.

Dalm SU, Sieuwerts AM, Look MP, Melis M, van Deurzen CH, Foekens JA, et al. (2015b). "Clinical Relevance of Targeting the Gastrin-Releasing Peptide Receptor, Somatostatin Receptor 2, or Chemokine C-X-C Motif Receptor 4 in Breast Cancer for Imaging and Therapy. Journal of Nuclear Medicine : official publication, Society of Nuclear Medicine 56: 1487-93.

FDA Guidance for Industry, Investigators, and Reviewers, "Exploratory IND Studies" (January 2006)

Ferlay J, Colombet M, Soerjomataram I, Dyba T, Randi G, Bettio M, Gavin A, Visser O, Bray F. Cancer incidence and mortality patterns in Europe: Estimates for 40 countries and 25 major cancers in 2018. Eur J Cancer. 2018 Nov;103:356-387.

Grootendorst MR, Cariati M, Pinder S, Kothari A, Douek M, Kovacs T, Hamed H, Pawa A, Nimmo F, Owen J, Ramalingam V, Sethi S, Mistry S, Vyas K, Tuch D, Britten A, Van Hemelrijck M, Cook G, Sibley-Allen C, Allen S, Purushotham A (2017) “Intraoperative Assessment of Tumor Resection Margins in Breast-Conserving Surgery using 18F-FDG Cerenkov Luminescence Imaging - A First-in-Human Feasibility Study”. Journal of Nuclear Medicine 58: 891–898.

Halmos G, Wittliff JL, Schally AV (1995). “Characterization of bombesin/gastrin-releasing peptide receptors in human breast cancer and their relationship to steroid receptor expression”. Cancer Research 55: 280-7.

Heckathorne E, Dimock C, Dahlbom M (2008). "Radiation dose to surgical staff from positron-emitter-based localization and radiosurgery of tumours". Health Physics 95: 220–226.

ICRP (1992). “Radiological Protection in Biomedical Research”. ICRP Publication 62. Ann. ICRP 22 (3).

ICRP (2007). "2007 Recommendations of the International Commission on Radiological Protection (Users Edition)". ICRP Publication 103 (Users Edition). Ann. ICRP 37 (2-4).

Ionising Radiations Regulations 2017, Approved Code of Practice and Guidance L121

Jeevan, R, Cromwell, DA, Trivella, M, Lawrence, G, Kearins, O, Pereira, J, van der Meulen, JHP (2012). "Reoperation rates after breast conserving surgery for breast cancer among women in England: retrospective study of hospital episode statistics". BMJ 345 e4505.

Jensen RT, Battey JF, Spindel ER, Benya RV (2008). “International Union of Pharmacology. LXVIII. Mammalian bombesin receptors: nomenclature, distribution, pharmacology, signaling, and functions in normal and disease states”. Pharmacol Rev. 2008 60(1):1-42.

Jurrius, P. A. G. T., Grootendorst, M. R., Krotewicz, M., Cariati, M., Kothari, A., Patani, N., Karcz, P., Nagadowska, M., Vyas, K. N., Purushotham, A., & Turska-d’Amico, M. (2021). Intraoperative [18F]FDG flexible autoradiography for tumour margin assessment in breast-conserving surgery: a first-in-human multicentre feasibility study. EJNMMI Research, 11(1), 28. https://doi.org/10.1186/s13550-021-00759-w

Liu, H, Carpenter, CM, Jiang, H, Pratx, G, Sun, C, Buchin, MP, Cheng, Z (2012). "Intraoperative imaging of tumours using Cerenkov luminescence endoscopy: a feasibility experimental study". Journal of Nuclear Medicine 53(10), 1579–84.

Mansel, RE, Fallowfield L, Kissin M, Goyal A, Newcombe RG, Dixon JM, Yiangou C, Horgan K, Bundred N, Monypenny I, England D, Sibbering M, Abdullah TI, Barr L, Chetty U, Sinnett DH, Fleissig A, Clarke D, Ell PJ (2006). "Randomized multicenter trial of sentinel node biopsy versus standard axillary treatment in operable breast cancer: the ALMANAC Trial". Journal of the National Cancer Institute 98, 599-609.

Merrill AL, Coopey SB, Tang R, McEvoy MP, Specht MC, Hughes KS, Gadd MA, Smith BL (2016). "Implications of New Lumpectomy Margin Guidelines for Breast-Conserving Surgery: Changes in Reexcision Rates and Predicted Rates of Residual Tumor". Ann Surg Oncol. Mar;23(3):729-34.

Moore, GE (1947). "Fluorescein as an Agent in the Differentiation of Normal and Malignant Tissues". Science 106(2745): 130-1.

Moran MS, Schnitt SJ, Giuliano AE, Harris JR, Khan SA, Horton J, Klimberg S, Chavez-MacGregor M, Freedman G, Houssami N, Johnson PL, Morrow M; Society of Surgical Oncology; American Society for Radiation Oncology (2014). "Society of Surgical Oncology-American Society for Radiation Oncology consensus guideline on margins for breast-conserving surgery with whole-breast irradiation in stages I and II invasive breast cancer". J Clin Oncol 10;32(14):1507-15.

Morgat C, MacGrogan G, Brouste V, Vélasco V, Sévenet N, Bonnefoi H, Fernandez P, Debled M, Hindié E (2017). "Expression of Gastrin-Releasing Peptide Receptor in Breast Cancer and Its Association with Pathologic, Biologic, and Clinical Parameters: A Study of 1,432 Primary Tumors". J Nucl Med. 2017 58(9):1401-1407.

Nalley C, Wiebeck K, Bartel TB, Bodenner D, Stack BC Jr. (2010). "Intraoperative radiation exposure with the use of (18)F-FDG-guided thyroid cancer surgery". Otolaryngol Head Neck Surg. 142(2): 281-3.

National Institute for Health and Care Excellence (NICE). (2009) "Early and locally advanced breast cancer: diagnosis and treatment". NICE guidelines [CG80]. February 2009 (Updated November 2015 without changes to margin width recommendation)

Nguyen, FT, Zysk, AM, Chaney, EJ, Kotynek, JG, Oliphant, UJ, Bellafiore, FJ, Boppart, SA (2009). "Intraoperative evaluation of breast tumour margins with optical coherence tomography". Cancer Research 69(22): 8790–6.

Olde Heuvel, J., Veen, B. J. D. W., Tuch, D. S., Vyas, K., Grootendorst, M. R., & Leitao, P. D. S. (2018). Performance evaluation of Cerenkov Luminescence Imaging versus Autoradiography. European Journal of Nuclear Medicine and Molecular Imaging, *45*(S1), pS252–pS253. https://doi.org/10.1007/s00259-018-4148-3

Performance evaluation of in vitro diagnostic medical devices. BS EN 13612:2002

Piert M, Burian M, Meisetschläger G, Stein HJ, Ziegler S, Nährig J, Picchio M, Buck A, Siewert JR, Schwaiger M (2007). "Positron detection for the intraoperative localisation of cancer deposits". European Journal of Nuclear Medicine and Molecular Imaging 34(10): 1534-44.

Pleijhuis, RG, Graafland, M, de Vries, J, Bart, J, de Jong, JS, van Dam, GM (2009). "Obtaining adequate surgical margins in breast-conserving therapy for patients with early-stage breast cancer: current modalities and future directions". Annals of Surgical Oncology 16(10): 2717–30.

Povoski SP, Sarikaya I, White WC, Marsh SG, Hall NC, Hinkle GH, Martin EW Jr, Knopp MV (2008). "Comprehensive evaluation of occupational radiation exposure to intraoperative and perioperative personnel from ^{18}F-FDG radioguided surgical procedures". Eur J Nucl Med Mol Imaging 35, 2026-2034.

Position paper on non-clinical safety studies to support clinical trials with a single microdose, June 2004, CPMP/SWP/2599/02/Rev 1)1

Reubi JC, Fleischmann A, Waser B, Rehmann R (2011). “Concomitant vascular GRP-receptor and VEGF-receptor expression in human tumors: molecular basis for dual targeting of tumoral vasculature”. Peptides 32(7):1457-62.

Reubi JC, Wenger S, Schmuckli-Maurer J, Schaer JC, Gugger M (2002). “Bombesin receptor subtypes in human cancers: detection with the universal radioligand (125)I-[D-TYR(6), beta-ALA(11), PHE(13), NLE(14)] bombesin(6-14). Clinical Cancer Research : an official journal of the American Association for Cancer Research 8: 1139-46.

Robertson, R, Germanos, MS, Manfredi, MG, Smith, PG, & Silva, MD (2011). "Multimodal imaging with (18)F-FDG PET and Cerenkov luminescence imaging after MLN4924 treatment in a human lymphoma xenograft model." Journal of Nuclear Medicine 52(11), 1764–9.

Toss MS, Pinder SE, Green AR, Thomas J, Morgan DA, Robertson JF, Ellis IO, Rakha EA (2017). “Breast conservation in ductal carcinoma in situ (DCIS): what defines optimal margins?”. Histopathology 70(5):681-692.

U.S. Department Of Health And Human Services, National Institutes of Health, National Cancer Institute (2009). "Common Terminology Criteria for Adverse Events v4.0. (v4.03: June 14, 2010)" NIH publication # 09-7473.

Van de Wiele C, Phonteyne P, Pauwels P, Goethals I, Van den Broecke R, Cocquyt V, et al (2008). “Gastrin-releasing peptide receptor imaging in human breast carcinoma versus immunohistochemistry”. Journal of nuclear medicine :

official publication, Society of Nuclear Medicine. 2008; 49: 260-4.

Verbruggen A, Coenen HH, Deverre JR, Guilloteau D, Langstrom B, Salvadori PA, Halldin C (2008). "Guideline to regulations for radiopharmaceuticals in early phase clinical trials in the EU." European Journal of Nuclear Medicine and Molecular Imaging 35(11): 2144-5

Vitug, AF, Newman LA (2007). "Complications in breast surgery. The Surgical Clinics of North America 87(2): 431–51

Vyas KN, Grootendorst M, Mertzanidou T, Macholl S, Stoyanov D, Arridge SR. et al. Flexible scintillator autoradiography for tumor margin inspection using 18F-FDG. Proceedings Volume 10478, Molecular-Guided Surgery: Molecules, Devices, and Applications IV; 1047811 (2018)

Wall BF, Haylock R, Jansen JTM, Hillier MC, Hart D, Shrimpton PC (2011). "Radiation Risks from Medical X-ray Examinations as a Function of the Age and Sex of the Patient." HPA-CRCE-028

Waser B, Eltschinger V, Linder K, Nunn A, Reubi JC (2007). “Selective in vitro targeting of GRP and NMB receptors in human tumours with the new bombesin tracer 177Lu-AMBA”. Eur J Nucl Med Mol Imaging 34(1): 95-100.
